# A Method to Culture GABAergic Interneurons Derived from the Medial Ganglionic Eminence

**DOI:** 10.3389/fncel.2017.00423

**Published:** 2018-01-08

**Authors:** Sira A. Franchi, Romina Macco, Veronica Astro, Diletta Tonoli, Elisa Savino, Flavia Valtorta, Kristyna Sala, Martina Botta, Ivan de Curtis

**Affiliations:** ^1^Cell Adhesion Unit San Raffaele Scientific Institute and San Raffaele University, Milan, Italy; ^2^Neuropsychopharmacology Unit, Division of Neuroscience, San Raffaele Scientific Institute and San Raffaele University, Milan, Italy

**Keywords:** GABAergic interneurons, medial ganglionic eminences, neurites, growth cones, Rac GTPases

## Abstract

Understanding the mechanisms guiding interneuron development is a central aspect of the current research on cortical/hippocampal interneurons, which is highly relevant to brain function and pathology. In this methodological study we have addressed the setup of protocols for the reproducible culture of dissociated cells from murine medial ganglionic eminences (MGEs), to provide a culture system for the analysis of interneurons *in vitro*. This study includes the detailed protocols for the preparation of the dissociated cells, and for their culture on optimal substrates for cell migration or differentiation. These cultures enriched in interneurons may allow the investigation of the migratory behavior of interneuron precursors and their differentiation *in vitro*, up to the formation of morphologically identifiable GABAergic synapses. Live imaging of MGE–derived cells plated on proper substrates shows that they are useful to study the migratory behavior of the precursors, as well as the behavior of growth cones during the development of neurites. Most MGE-derived precursors develop into polarized GABAergic interneurons as determined by axonal, dendritic, and GABAergic markers. We present also a comparison of cells from WT and mutant mice as a proof of principle for the use of these cultures for the analysis of the migration and differentiation of GABAergic cells with different genetic backgrounds. The culture enriched in interneurons described here represents a useful experimental system to examine in a relatively easy and fast way the morpho-functional properties of these cells under physiological or pathological conditions, providing a powerful tool to complement the studies *in vivo*.

## Introduction

The γ-aminobutyric acid (GABA)-ergic interneurons are modulators of brain function essential to keep the balance between excitation and inhibition (Gelman and Marín, 2010). If these cells or their functions are altered during development, the balance is affected, and this may cause various cognitive and neurological diseases such as schizophrenia, epilepsy, and hyperactivity disorders (Kitamura et al., 2002; Lewis et al., 2005; Orekhova et al., 2007; Lawrence et al., 2010; Brooks-Kayal, 2011; Sebe and Baraban, 2011; Won et al., 2011). GABAergic interneurons represent about 20% of cortical/hippocampal neurons. Most cortical and hippocampal GABAergic cells originate from ganglionic eminences, transitory embryonic structures of the ventral telencephalon (Wonders and Anderson, 2006). The medial ganglionic eminence (MGE) is one of these regions that gives rise to important classes of cortical and hippocampal GABAergic cells (Tricoire et al., 2011; Wamsley and Fishell, 2017). Interneurons born in the MGE subsequently migrate tangentially along the marginal zone, the subventricular zone, and the subplate. Switching from tangential to radial migration, they reach and populate different layers within the cortex and the hippocampus (Guo and Anton, 2014). In their migration interneuron precursors are guided to reach their final destination by extracellular cues including motogenic factors and neurotrophins (Hernández-Miranda et al., 2010). Once they have reached their final destination, they make synaptic connections with excitatory and other inhibitory neurons. The differentiation and maturation of interneurons therefore involve sequential processes including migration, the outgrowth, and branching of axons and dendrites, and finally the formation of inhibitory synapses.

We have recently set up an *in vitro* system highly enriched in interneurons obtained by dissociated MGEs isolated from E14.5 mice. The differentiation of the dissociated MGE-derived precursors is evident already after 6 days *in vitro* (DIV6). By DIV6 virtually all the MGE-derived precursors develop into neurons positive for the neuronal markers TuJ1, MAP2, and Tau1, and 70% of these cells are already positive for GABA. This *in vitro* system has been recently successfully utilized for the morphological analysis of the differentiation, neuritogenesis and synapse formation in GABAergic cells *in vitro*, in the context of the study of the implication of Rac proteins and an associated protein network in these processes, by evaluating effects of silencing and of mutant overexpression on various parameters, including neuritic length and branching, the morphology and dynamics of growth cones, and the subcellular distribution of the endogenous proteins under study (Franchi et al., 2016).

Here we describe in details the protocols set up to reproducibly obtain cultures of MGE-derived cells, by presenting the optimization of these cultures in terms of substrate and conditions for the transfection of plasmids.

We have organized this methodological study in different sections within the Results and Discussion. For each section we first describe and discuss the issues addressed during the setup of the method, and then describe the detailed finalized protocol. Reagents, compositions of the main solutions, and general methods are included in Materials and Methods. The results include the description of the setup of the mouse MGE–derived cultures: dissociation of MGE–derived cells, plating and optimization of the extracellular substrates for morpho-funtional analysis of differentiation and for cell migration; characterization of the MGE–derived cultures, including methods for immunochemical analysis; examples of the use of the MGE–derived dissociated cell cultures for the analysis of the differentiation and migration of a strain of KO mice; effects of BDNF; setup of the transfection protocol.

Although some studies have used MGE explants or short-term cultures of MGE-derived cells to address the role of different extracellular cues and their receptors in the migration and development of interneurons (Pozas and Ibáñez, 2005; Cobos et al., 2007; Zimmer et al., 2008; Rudolph et al., 2010; Li et al., 2012; Villar-Cerviño et al., 2015), detailed protocols and optimization of conditions to obtain longer term cultures, or to transfect these cells are not available. The methods to setup cultures of MGE–derived interneurons described here may allow the morpho-functional analysis of GABAergic cells *in vitro*, and the exploration of the machinery underlying their development and function.

The detailed procedures described in this methodological study have been optimized to specifically culture MGE-derived cells. The study includes the definition of the conditions for interneuron cultures by using the optimized combination of specific substrates and BDNF, and the practical detailed indication of the protocols needed to obtain viable cultures. The study also includes the description of cultures on distinct specific substrates adapt to investigate either the migration or the maturation of interneurons, and shows the development of different types of GABAergic cells *in vitro*, as defined by the use of markers specific for distinct interneuron subtypes. We have identified the procedures that allow to reproducibly obtain longer-term interneuron cultures, which are relevant to the study of later events in interneuron development, such as synaptogenesis and synaptic function. Moreover, we have setup and described in details the conditions for proper transfection of the cultured MGE-derived cells. This is an important aspect of the study, since we found that the application of previously reported transfection procedures were inappropriate for the transfection of MGE-derived cells, due to either insufficient transfection efficiency, or to the heavy loss of cells following the transfection procedures that are in use with other types of cells and neurons.

## Materials and methods

In this section are listed reagents and solutions, and described basic techniques, while the development and description of the detailed protocols specific for the preparation and use of MGE-derived cultures are described at the end of each section of the Results and Discussion.

### Reagents and solutions

All solutions are prepared with double distilled (dd) water. Sterile materials are used for dissections and cell cultures.

– 69% nitric acid (VWR, highly corrosive, to be used under a fume hood; avoid pouring/mixing with water).
– round 13 mm diameter glass coverslips (Zeus super).
– 10 cm petri dishes, Sterilin (Thermo Scientific).
– Poly-L-lysine (PLL, Sigma, P2636). Prepare a stock solution at 1 mg/ml in 0.1 M borate buffer pH 8.5; filter solution with a syringe through a 0.2 μm pore filter; aliquot and store the stock solution at −20°C. Thaw each aliquot two times, then discard.
– PBS; PBS^+^, with Ca^2+^ (1 mM) and Mg^2+^ (0.5 mM).
– TBS: 150 mM NaCl, 20 mM Tris-HCl pH 7.5.
– Laminin-1 (LN) stock (mg/ml range concentration) in 100 mM MOPS, 80 mM CaCl_2_, stored in 25 μl aliquots at −80°C. Thaw each aliquot two times, then discard.
– 1 M HEPES buffer solution (Life Technologies, 15630-056).
– HBSS/HEPES: 100 ml Hanks' Balanced Salt Solution (HBSS; Life Technologies, cod. 14175-137) supplemented with 1 ml of 1 M HEPES (10 mM final).
– Leibovitz's L-15 medium (Life Technologies, 21083-027).
– Neurobasal Medium (Life Technologies, 21103-049).
– 10 mg/ml DNase (Calbiochem, DN25) stock solution, stored at −20°C in 300 μl aliquots; can be thawed several times.
– 2.5% trypsin solution (Gibco, 15090-046) to be stored in 70 μl aliquots at −20°C; discard excess after thawing.
– 10 μg/ml BDNF (PeproTech, 450-02) in PBS, 0.1% BSA; store 20 μl aliquots at −80°C; freeze and thaw twice, then discard.
– 4′,6-Diamidine-2′-phenylindole dihydrochloride (DAPI, from Sigma).

### Culture media

– Maintenance medium: to 50 ml of Neurobasal medium (Life Technologies, 21103-049) add 1 ml B-27 supplement (Life Technologies, 17504044) and 0.5 ml GlutaMAX (Life Technologies, 35050-061).
– Plating medium: to 22.5 ml of Neurobasal medium (Life Technologies, 21103-049) add 2.5 ml fetal bovine serum (10% final; Gibco/Invitrogen, 10270), 0.5 ml B-27 Supplement (Life Technologies, 17504-044), 0.25 ml GlutaMAX (Life Technologies, 35050-061).

### Animals

The MGE-derived cultures were obtained from C57BL/6J (WT), or from Rac3KO mice (Corbetta et al., 2005, 2009). All animal procedures were performed in accordance with the IACUC of the San Raffaele Scientific Institute, in full compliance with the national regulations (D.L. n 116, G.U. suppl. 40, 1992 February 18, circular Nr. 8, G.U., 1994 July 14) and with the international agreements in force (EEC Council directive 86/609, OJ L 358, 1 DEC 12, 1987; NIH Guide for the Care and use of Laboratory Animals, U.S. National Research Council, 1996).

### Cell lines

COS-7 cells were cultured in Dulbecco's Modified Eagle's Medium containing 4.5 g/l glucose, 10% FetalClone III (Euroclone). NIH-3T3 and HeLa cells were cultured in Dulbecco's Modified Eagle's Medium containing glucose 4.5 g/l, 10% fetal bovine serum (Euroclone). MDA-231 cells were growth in Dulbecco's Modified Eagle's Medium:F12 at 1:1 with 10% fetal bovine serum.

### Plasmids

Plasmids pEGFP-N1 and RFP-LifeAct are from Clontech Laboratories. The pSUPER.neo–GFP plasmid (VEC-PBS-0006) used as vector system for the expression of the control short interfering RNA shLuc was as described (Franchi et al., 2016).

### Antibodies

The primary antibodies used for immunofluorescence or immunoblotting include the mouse mAbs anti-ERC1 IgG2a (Abcam), anti-FLAG IgG1 (Sigma), anti-GAD67 IgG2a (Chemicon Millipore), anti-Gephyrin IgG1 (Synaptic Systems), anti-GIT/PKL (BD Bioscience); anti-MAP2 IgG1 (Sigma); anti-NeuN IgG1 (Millipore), anti-Tau1 IgG2a (Chemicon Millipore), anti-tubulin (Sigma); anti-TuJ1 IgG2a (Covance); the rat mAb anti-somatostatin (Millipore); the rabbit pAbs anti-calbindin (Swant), anti-FLAG (Sigma), anti-GABA (Sigma); anti-GFP (Molecular Probe, Invitrogen), anti-HA (BioLegend), anti-HA (BABCO), anti-liprin-α1 (ProteinTech), anti-MAP2 (Santa Cruz), anti-parvalbumin (Swant), anti-βPIX si255 (Paris et al., 2003; Za et al., 2006), anti-Rac3 (Proteintech), anti-VGAT (Synaptic Systems), anti-neuronal nitric oxide synthase (Millipore), anti-calretinin (Millipore); chicken pAb anti-GFP (Abcam); goat pAbs anti-GIT1 (Santa Cruz), anti-PAK3/βPAK (Santa Cruz), anti-parvalbumin (Swant).

Secondary antibodies for immunofluorescence are Alexa Fluor A488/568/647-conjugated antibodies (Invitrogen) including: donkey anti-goat IgG; donkey anti-rabbit IgG; donkey anti-rat IgG; goat anti-rabbit IgG; goat anti-chicken IgG; goat anti-mouse IgG; goat anti-mouse IgG1; goat anti-mouse IgG2a. TRITC-conjugated phalloidin (Sigma).

Secondary antibodies used for immunoblotting include donkey anti-rabbit IgG HRP-conjugated (GE Healthcare); sheep anti-mouse IgG HRP-conjugated (GE Healthcare); rabbit anti-goat IgG HRP-conjugated (Southern Biotech).

### Imaging and migration assays

For the analysis of random migration, dissociated MGE-derived cells were plated on Matrigel–coated coverslips (100,000 cells/well), and analyzed for migration at DIV2. During image acquisition, cells were maintained at 37°C in a CO_2_-controlled chamber, and time-lapse images were acquired at a Zeiss Axiovert S100 microscope equipped with 20x lens. Cell tracking on images was performed with the Manual Tracking plugin of ImageJ. Randomly chosen well-isolated cells were tracked for 14 h with 95 time points (about 9–min intervals). The analysis was performed with the Chemotaxis Tool plugin of ImageJ.

For high resolution time-lapse imaging, cells were maintained at 37°C in a CO_2_—controlled chamber, and images acquired at the indicated intervals at a confocal Leica SP8 using a 63 × 1.4 NA oil immersion objective.

For the quantification of the expression of neuronal and GABAergic markers, immunofluorescence images from cultures with WT and Rac3KO MGEs at different DIVs were acquired at a Zeiss microscope with 20x lens, and analyzed with ImageJ. For each image, the background signal was subtracted (including background from negative controls from coverslips treated with secondary antibodies only).

### Sholl analysis

For Sholl analysis images are acquired on an upright Zeiss Microscope with a 20x NA 0.50 objective and analyzed using the public domain NIH Image software ImageJ. Transfected GABAergic interneurons identified by GFP staining are overlaid by a mask consisting of 11 concentric circles of gradually increasing radius (from 20 to 120 μm), centered at the centroid of the cell body, drawn with the dedicated ImageJ plugin. The number of intersections between the rings and the neurites is scored manually. Only neurites with a length of at least 10 μm are considered. Data are analyzed by the Student's *t*-test. Differences were considered significant for *p* < 0.05.

### Immunochemical analysis

Cell lines, E14.5 mouse MGEs, and MGE-derived cultures were lysed with 0.5% Triton X-100, 150 mM NaCl, 20 mM Tris-HCl pH 7.5; 2 mM MgCl_2_, 1 mM sodium orthovanadate, 10 mM sodium fluoride and anti-proteases (1x Complete, Roche). Lysates were clarified by centrifugation, and protein concentration was determined through the Bradford protein assay (BIO-RAD Laboratories). Lysates were subjected to SDS-PAGE on acrylamide gels and used for immunoblotting. Primary antibodies were detected with HRP-conjugated secondary antibodies and revealed by ECL (Amersham Biosciences).

### Statistical analyses

Data are presented as the mean ± SEM throughout experiments. All statistical analyses were performed using Excel or Prism. Differences were tested using Student's *t-*test; *p* < 0.05 was considered significant. Statistical significance was presented in figures and throughout the text in the following manner: ^*^p < 0.05; ^**^p < 0.01; ^***^p < 0.001.

## Results and discussion

### Dissection of E14.5 MGEs and optimization of the culture conditions of dissociated cells

Here we describe the conditions tested and the protocols to obtain reproducible cultures of developing GABAergic interneurons up to the formation of morphologically identifiable GABAergic synapses. In this section we first describe the protocol for the isolation of MGEs from E14.5 mouse embryos; then present the detailed protocols required to set up the cultures on coverslips for morphological and functional analysis, or on tissue plastic for biochemical analysis; and finally we describe the conditions tested to optimize the culture of MGE-derived dissociated cells by using different extracellular substrates.

#### Protocol for the dissection of the MGEs from E14.5 mouse embryos

**Figure 1:**
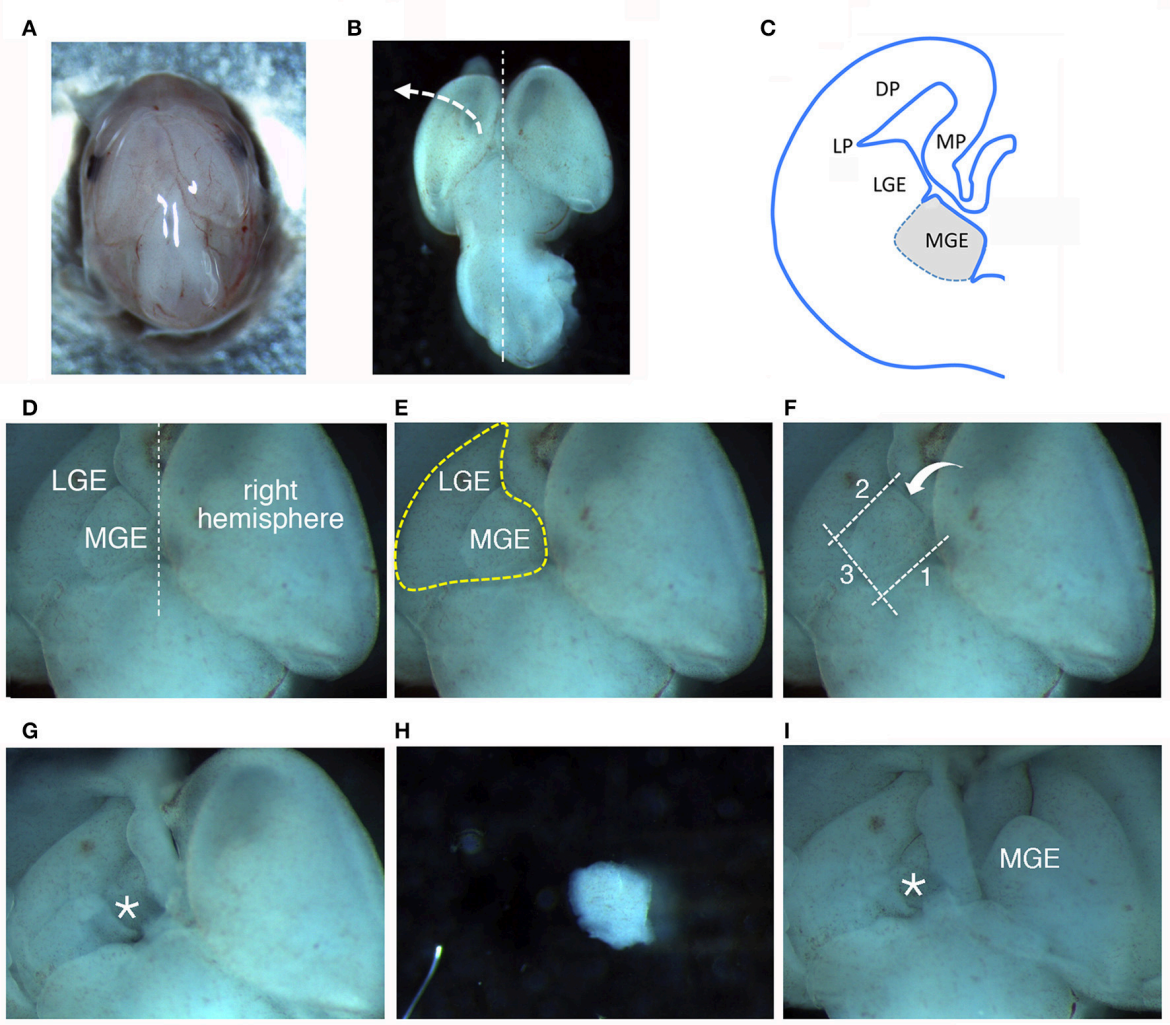
Dissection of MGEs from E14.5 mouse embryos. **(A)** View from the top of a E14.5 head. **(B)** View from the top of a E14.5 brain. **(C)** Scheme of a coronal hemisection of E14.5 mouse brain showing the lateral ganglionic eminence (LGE) and medial ganglionic eminence (MGE). DP, dorsal pallium; LP, lateral pallium; MP, medial pallium. **(D–F)** The same image is represented to remark different aspects described in the main text: **(D)** MGE exposed after lifting and moving aside the left cortical hemisphere; **(E)** Heart-like subcortical structure including MGE and LGE; **(F)** dotted lines 1 to 3 indicate in order the cuts made with the microscalpel to isolate the MGE; the bent arrow indicates the side for the last cut. **(G)** Brain as in **(D–F)** after removal of the MGE. The area where the MGE was present is indicated by the asterisk. **(H)** Isolated MGE. **(I)** Same brain as in **(G)** after lifting on the side the right cortical hemisphere to expose the second MGE.

– Prepare: two 10 cm diameter cell culture dishes with 15 ml/dish of HBSS/HEPES to collect the embryos (in the first dish), and the heads (in the second dish; see below); 6 cm dishes (one per head dissection) with 4 ml of L-15 medium for the dissections; a 3.5 cm dish with 3 ml of HBSS/HEPES to collect the MGEs.
– All steps, with the exception of dissection itself, are performed on ice.
– Extract the embryos from the placenta and rinse them in the first of the two 10 cm cell culture dishes with HBSS/HEPES on ice. Then transfer the embryos to the second 10 cm dish with HBSS/HEPES on ice.
– Separate the head from the rest of one embryo with tweezers.
– Transfer the head to a 6 cm dish with 4 ml of L-15 medium; move the dish under a stereomicroscope and work at room temperature (RT) (Figure 1A); remove the layers surrounding the brain tissue avoiding to damage the brain structures (Figure 1B). The two MGEs lie under the two cerebral hemispheres (Figure 1C).
– Hold the brain by the pons with tweezers, to manipulate more easily the tissue during the dissection.
– Using a second pair of tweezers:
  - lift and move to the side one of the cerebral hemispheres (Figure 1B), to expose the underlying GEs. A heart-like structure with two lobes (including MGE and LGE) will appear (Figure 1D): the MGE is the lobe closer to the midline between the two hemispheres (Figure 1E).
  - With a microscalpel, make a first cut along line 1 (Figure 1F, line 1).
  - Make a second cut parallel to the first one, between the MGE and the LGE (Figure 1F, line 2);
  - then make a third cut perpendicular to the first two cuts (Figure 1F, line 3);
  - and a final spoon-like cut passing under the MGE (Figure 1F, bent arrow), to separate it from the rest of the cortex (Figures 1G–H).
  - Transfer the isolated MGE to the 3.5 cm dish with 3 ml of ice-cold HBSS/HEPES.
  - Repeat steps (a–f) to remove the MGE under the second hemisphere (Figure 1I).
– Use a new 6 cm petri dish with 4 ml of L-15 medium for the dissection of each embryo.

#### Protocol for the dissociation and culture of MGE-derived cells

Conditions for the development of cells with neuronal morphology were identified by culturing MGE–derived cells on PLL– and LN–coated substrates. Here below we describe the detailed protocol to obtain reproducible cultures.

##### Preparation of glass coverslips

– 13 mm round glass coverslips are loaded in porcelain racks.
– Place racks in a glass container.
– Under a fume hood, pour 69% nitric acid in the glass container by using a pipettor with 25 ml glass pipette, until the racks with coverslips are fully covered.
– Cover the container with a glass cover and leave overnight under the fume hood on.
– The next morning lift the racks with tweezers, and immerse them into a glass container already filled with dd water (caution: do not pour water on the racks wet with nitric acid, it may cause explosion).
– Continue the washings by using a total of 2 lt of dd water: about 10 washes of 200 ml water, 40 min per wash.
– The nitric acid left in the first glass container can be used for a second round of coverslips up to the next day, otherwise discard it properly.
– After the last wash, the coverslips are transferred to an empty 2 lt beaker and dried for 2 h on a hot plate (do not heat above 150°C), or overnight at RT.
– Coverslips are stored in a 10 cm glass petri dish, and sterilized in an oven at 180°C.

##### Coating of glass coverlips with PLL and LN

Coating of coverlips with PLL and LN, which is the best substrate identified by us to culture dissociated mouse E14.5 MGE–derived cells, as described in details in the next section. Important note: a particular care has to be placed to avoid that coverslips dry after coating with PLL and LN. For this, removal of solutions has to be made rapidly before addition of the next solution. This is important to avoid that proteins like LN lose their biological activity.

– The morning of the dissection coverslips are transferred to 10 cm Sterilin petri dish (up to 12 coverslips of 13 mm diameter per petri dish). This type of dishes favors the washing of coverslips by surface tension of the liquid on the coverslips, avoiding dispersion of solutions that may cause uneven treatment and drying of the surface of the coverslips.
– Prepare 200 μg/ml PLL (100 μl/coverslip, by diluting 1:5 in dd water the 1 mg/ml stock of PLL).
– Place 100 μl of PLL on each coverslip, paying attention to cover the whole surface; incubate the coverslips in the petri with the lid for 2 h in a 37°C incubator.
– Remove the PLL by aspiration with a P1000 pipette; wash each coverslip for 5 min with 300 μl of PBS; pay attention to distribute the PBS over the whole surface, avoiding overflowing of the solution out of the coverslips.
– Remove the PBS with a P1000 pipette; wash once for 5 min with TBS, in preparation of the coating with LN prepared in MOPS/CaCl_2_. If the LN stock is in a buffer without calcium, PBS instead of TBS can be used for the second wash.
– Prepare a humidified chamber by using a 15 cm plastic petri dish with wet absorbent paper coated by parafilm, and sterilized by leaving it overnight under UV light in a laminar flow hood.
– Place 30 μl drops of 20 μg/ml LN (stock diluted with TBS) on the sterilized parafilm in the humidified chamber.
– Draw the washing solution from the coverslips with a P1000 pipette; use a vacuum set for suction prepared under the laminar flow hood to remove excess liquid from coverslips (to avoid dilution of the LN solution).
– After removing the excess washing buffer, place each coverslip upside down on one of the 30 μl drops of 20 μg/ml LN on parafilm in the humidified chamber and cover with the lid; incubate for 2 h in 37°C incubator.
– By using a P200 pipette, gently inject 200 μl of PBS under each coverslip to lift them, and move them back with tweezers to the Sterilin petri dish; wash each coverslip 3 times for 5 min each with 300 μl/coverslip of PBS.
– Remove the PBS with a P1000 pipette after each wash.
– At the end of the last wash, aspirate the liquid by vacuum under the laminar flow hood from the uncoated side of each coverslip, while using a P200 pipette to gently remove excess liquid from the edge of the coated side of each coverslip, so that most liquid is removed from the coverslips, but avoiding drying of the coated surface. This is important for two reasons: on one side the procedure avoids loss of biological activity by the coating molecules that may be induced by drying the coated surface; on the other side careful removal of excess buffer avoids small variations in the composition of the culture medium that may affect the cultures.
– Move each coverslip to a well with 300 μl of maintenance medium in a 24-well dish. Leave the plate in the 37°C incubator until cell plating.

##### Coating of glass coverslips with matrigel

Matrigel appears to be the best coating substrate on glass coverslips to follow migration in DIV2/DIV3 cultures of dissociated MGE–derived cells. The development of interneurons on Matrigel seems to occur more slowly with respect to PLL/LN. Although at early DIVs Matrigel is not as efficient as PLL and LN in promoting neurite outgrowth, we found that it facilitates long-term cultures compared to PLL and LN. Interneurons are maintained in culture on Matrigel until DIV15–DIV20 without BDNF.

– Place coverslips on 30 μl drops of 2.5 mg/ml Matrigel diluted in L15 medium, on sterilized parafilm (by UV o.n.) in a humid chamber.
– After 1.5 h at RT coverslips are lifted by adding L15 medium, and transferred to a 24-well plate with 300 μl of maintenance medium/well.
– Wait for at least 1 h to hydrate before adding the cells.

##### Dissociation of cells from isolated MGEs

– Under a laminar flow hood, collect the MGEs from the 3.5 cm dish with 3 ml of ice-cold HBSS/HEPES (see Protocol for the Dissection of the MGEs from E14.5 Mouse Embryos Above) with a pipettor and a 2 ml stripette, by aspirating them with the smallest possible liquid volume, and transfer them to a 15 ml sterile Falcon plastic tube (BD Biosciences). It is important to keep the MGEs concentrated within the tip of the stripette, since they tend to stick to its wall, making their recovery very difficult if not impossible when they stick away from the tip. Then transfer the remaining HBSS/HEPES from the dish to the 15 ml falcon tube to a final volume of 3 ml.
– Add to the 3 ml MGE suspension, 30 μl of 10 mg/ml DNase (100 μg/ml final), and 60 μl of 2.5% trypsin (0.05% final).
– Incubate 15 min in 37°C incubator, with the 15 ml Falcon tube standing.
– The enzymatic digestion is stopped by adding 5 ml of warm plating medium.
– Centrifuge for 5 min at 90 g, RT.
– Aspirate the supernatant with a 5 ml pipette; add 2 ml (for cells from 6–12 MGEs) or 3 ml (for cells from more than 12 MGEs) of maintenance medium; mechanically dissociate the tissue with 20 passages (i.e., 10 times up and down) through a P1000 pipette tip; resuspend gently, without friction against the tube wall; continue by resuspending with 12 passages (i.e., 6 times up and down) with a pipettor with a 2 ml stripette carrying a pipette yellow tip at the end, with moderate friction during pipetting, obtained by placing the yellow tip against the wall of the 15 ml tube without pressing.
– Count the cells: mix 80 μl of maintenance medium with 10 μl of cell suspension and 10 μl of Trypan Blue; put 10 μl of this mix on each of two sides of a Neubauer chamber to count cells. As an indicative example, from a dissection from 2 pregnant mice with a total of 11 E14.5 embryos and the collection of 22 MGEs, 7.2 × 10^6^ cells resuspended in 3 ml of maintenance medium were obtained. On average, we calculated that approximately 400,000 cells per MGE can be obtained after dissociation with trypsin. These cells can be used for morpho-functional or biochemical analysis, as detailed in the next sections.

##### Cell plating

Plate cells according to the different requirements. For plating cells on PLL/LN coated coverslips prepared in 24-well dishes (as described above):

– for morphological analysis: between 25.000 to 50.000 cells per coverslip in 800 μl of maintenance medium.
– for transfection: not less than 100.000 cells per coverslip in 800 μl of maintenance medium.
– for biochemistry: typically 1.5–3 × 10^6^ dissociated MGE–derived cells (obtained from about 4-7 E14.5 MGEs) are plated in each 3.5 cm diameter dish with a total of 1.5 – 2.5 ml of maintenance medium per dish. Dishes can be coated with 100 μg/ml PLL for 2 h and then washed twice for 5 min each with PBS before adding the cells.

##### Cultures

When required (see next sections), add BDNF at DIV1 following this procedure:

– Remove 600 μl of medium, leaving 200 μl of medium in each well of the 24-well plate.
– Pool the aliquots of medium from different wells in a 15 ml sterile Falcon tube.
– Dilute the stock of BDNF (10 μg/ml) in the collected conditioned medium, to a final concentration of 100 ng/ml. It is important to avoid substituting the old medium with fresh non-conditioned medium, since this may remove factors secreted by interneurons that are important for their long-term survival.
– Add to each well 200 μl of medium with BDNF, to have a final volume of 400 μl of medium with 50 ng/ml BDNF.
– For cultures prepared in 3.5 cm diameter dishes to be used for biochemical analysis, at DIV1 remove 1.75 ml of the 2.5 ml of medium from each dish, dilute BDNF to 100 ng/ml of conditioned medium, and add back 0.75 ml of conditioned medium with BDNF to each dish, to a final volume of 1.5 ml of medium with 50 ng/ml BDNF.

#### Optimization of the extracellular substrates for culturing E14.5 MGE–derived cells

Different cell types express on their surface distinct sets of adhesive receptors for different extracellular ligands. To identify conditions that would allow the adhesion and development of interneurons with a differentiated neuritic network, we have tested a number of common extracellular matrix (ECM) proteins as substrates for the cultures.

**Figure 2:**
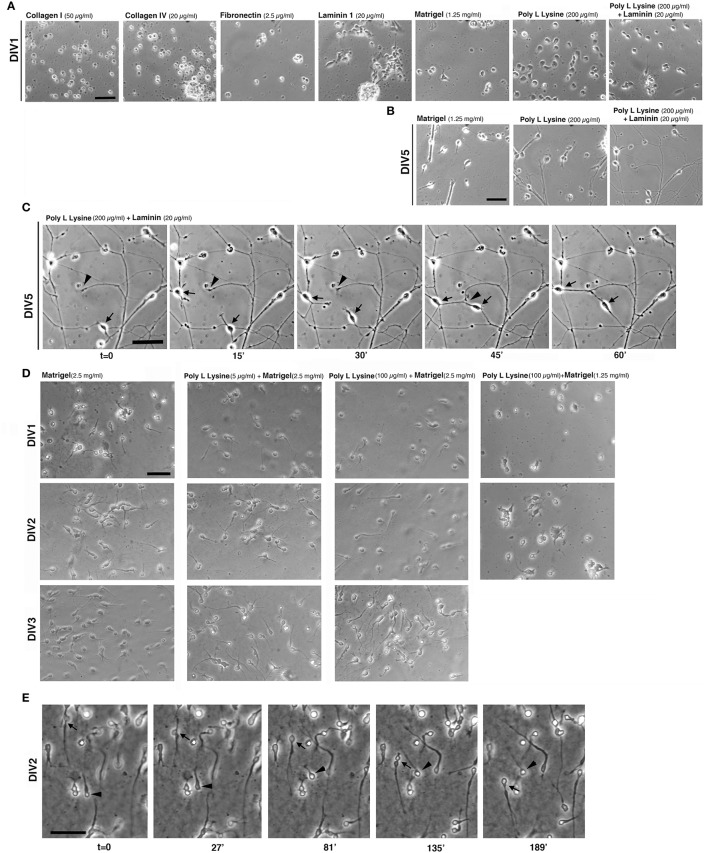
Extracellular substrates for MGE–derived cultures. **(A,B)** Images from living MGE-derived cells plated on coverslips coated with the indicated substrates and cultured for 24 h **(A)** or 5 days **(B)**. Coatings of coverslips were as follows: Matrigel (1.25 mg/ml in L-15 medium, 40 μl/coverslip), fibronectin (2.5 μg/ml), or laminin (20 μg/ml in TBS) o.n. at 4°C; poly L lysine (200 μg/ml) o.n. in 37°C incubator. Collagen I (50 μg/ml in 0.02 N acetic acid) or collagen IV (20 or 40 μg/ml in 0.05 N HCl) 3 h at RT; poly L lysine (200 μg/ml) o.n. in 37°C incubator followed by coating with laminin (20 μg/ml in TBS) for 3 h at RT. After washing the coverslips three times with PBS, 30,000 cells per coverslips were plated. **(C)** DIV5 MGE-derived cells plated on poly L lysine and laminin; part of the field in the right panel of **(B)** is shown. Cells were imaged by phase contrast with Zeiss Axiovert S100 microscope equipped with 32x lens. Arrows and arrowheads point to two motile cells and a growth cone, respectively. **(D)** Test to set up conditions for MGE-derived cell migration. Images are snapshots from time-lapses of living MGE-derived cells at DIV1-DIV3 plated on coverslips coated with the indicated substrates. **(E)** Time-lapse analysis of MGE–derived cell migration. Coverslips were coated for 1.5 h at RT with 30 μl of 2.5 mg/ml Matrigel, placed in 24-wells dish and rehydrated for 1 h with 300 μl of maintenance medium (see Experimental Procedures). 95,000 E14.5 MGE–derived cells were added to each well. At DIV2 migration was followed by phase contrast with a Zeiss Axiovert S100 microscope Zeiss microscope with 20x lens. Images are frames from the time lapse. Arrow and arrowhead point to two migrating cells. Bars, 50 μm.

Tests were performed by coating glass coverslips with purified ECM components at concentrations taken from available protocols commonly used to culture non-neuronal cells or other types of neurons. The first set of tests included coating of coverslips with collagen I (50 μg/ml), collagen IV (20 and 40 μg/ml), fibronectin (FN, 2.5 μg/ml), or laminin 1 (LN, 20 and 40 μg/ml). In addition we tested coatings with PLL (up to 200 μg/ml), PLL and LN (200 μg/ml PLL coated with 20 μg/ml laminin-1), or Matrigel™ (1.25 mg/ml), a complex extracellular matrix that supports growth and differentiation of different cell types (Kleinman and Martin, 2005). Cells were analyzed for their morphology after 24 h in culture (DIV1, Figure 2A). MGE-derived cells did not attach or attached poorly on some substrates, including collagens I and IV, FN, and LN. On LN cells were motile but tended to form aggregates, probably due to poor cell-substrate adhesion.

On 1.25 mg/ml Matrigel MGE-derived cells attached and were motile; cells often formed clusters with a tendency to detach by DIV2-DIV3 (not shown). When adhesion to the substrate was insufficient, MGE–derived cells adhered to each other by cell-cell mediate mechanisms (Supplementary Figure 1), as detected by an aggregation assay (Kwon et al., 2000).

On 200 μg/ml PLL interneurons attached well and were poorly motile at DIV1. On substrates coated with a combination of PLL (200 μg/ml) and LN (20 μg/ml) MGE-derived cells attached and spread. At DIV1 cells were more motile that on PLL alone. Analysis at DIV5 (Figure 2B) showed that cells plated on PLL and LN developed a more extended neuritic network compared to cells on Matrigel or on PLL alone, where cells tended to form large clusters. Time-lapse imaging on DIV5 cultures showed motility of both cells and growth cones on PLL and LN, both contributing to the formation of the neuritic neuronal network (Figure 2C).

Since cells on Matrigel appeared more motile (not shown), we next tested some combinations of Matrigel with or without PLL to optimize the substrate for the analysis of MGE-derived cell migration (Figure 2D). Although the presence of PLL with a higher concentration of Matrigel (2.5 mg/ml) appeared to improve early adhesion of cells at DIV1 compared to DIV1 cells on Matrigel alone, no obvious differences were detectable at DIV2 and DIV3. On the other hand, decreasing the concentration of Matrigel (1.25 mg/ml) caused increased cell aggregation (Figure 2D). Therefore, DIV2/DIV3 MGE-derived cells plated on 2.5 mg/ml Matrigel can be useful for the analysis of their motility (Figure 2E), while plating on PLL and LN represents the optimal condition among those tested for the analysis of neuritogenesis *in vitro* (see next sections).

### Morphological and biochemical analysis of MGE–derived cultures

Dissociated MGE–derived cells may be used for either morpho-functional analysis, or for immunochemical analysis. Following are the protocols for immunofluorescence and immunoblotting on cultures of dissociated MGE–derived cells, as well as some examples relative to the use of either approach.

#### Protocol for the morphological analysis of MGE–derived cells by immunofluorescence

##### Solutions

– PBS; PBS^+^.
– 1.44 M sucrose, aliquots stored at −20°C.
– 100 mM EGTA, aliquots stored at −20°C.
– 500 mM MgCl_2_ stored at 4°C.
– 240 mM Na-phosphate buffer pH 7.4, prepared as follow: to 400 ml of 240 mM Na_2_HPO_4_ add slowly about 100 ml of 240 mM NaH_2_PO_4_ up to pH 7.4.
– High salt solution: 500 mM NaCl, 20 mM Na-phosphate buffer pH 7.4.
– Permeabilization buffer 2x; for 3 ml mix:
  - 1 ml of goat serum (heat inactivated by 30 min incubation in 56°C waterbath).
  - 180 μl 10% (w/v) Triton X-100 (final 0.6%).
  - 500 μl of 240 mM Na-phosphate buffer pH 7.4 (final 40 mM).
  - 54 μl of 5 M NaCl (final 90 mM).
  - 1266 μl of dd water.
– Goat serum dilution buffer 2x; for 3 ml mix:
  - 1 ml heat inactivated goat serum.
  - 500 μl of 240 mM Na-phosphate buffer pH 7.4 (final 40 mM).
  - 54 μl 5 M NaCl (final 90 mM).
  - 1446 μl of dd water.
– Anti-bleaching solution; for 10 ml:
  - 1 ml of 10x PBS (from Gibco or made in the lab).
  - 2 ml dd water 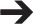
cool to 0-4°C.
  - Dissolve with the help of a P1000 pipette 7 mg of phenyl ethylenediamine (Sigma, P6001) in a darkened 15 ml Falcon plastic tube.
  - Add 7 ml glycerol to 10 ml final volume.
  - Mix on a rotating wheel in the cold room for 15 min.
  - Store the solution at −20°C; discard when it turns brown.
– Alternatively, the Dako Fluorescent Mounting Medium (Dako, Agilent Technologies) can be used. This mounting medium does not have glycerol and needs to be added carefully to avoid displacing the cells on coverslips. No evident differences between the two mounting agents have been observed on the preservation of the neuronal morphology and on the fluorescent signal after immunostaining with the antibodies that have been tested.
– Paraformaldehyde stock solution (16%), to be prepared under a fume hood as follows: add 16 g of paraformaldehyde to 90 ml of dd water in a beaker; heat to ≤55°C; add 100 μl of 1 N NaOH to complete the solubilization of paraformaldehyde. Once the paraformaldehyde is dissolved, neutralize the pH with 100 μl of 1 N HCl, then add 10 ml di 10x PBS^+^. Cool and filter through 0.46 μm bottle filter; make 3 ml aliquots in 15 ml Falcon plastic tubes and store at −20°C.

##### Fixation of interneurons

To prepare a 4% paraformaldehyde solution for fixation, thaw an aliquot of 3 ml of 16% paraformaldehyde and add:

- 1 ml of 1.44 M sucrose (from stock stored at −20°C; final 120 mM).
- 360 μl of 100 mM EGTA (from an aliquot stored at −20°C; final 3 mM).
- 48 μl of 500 mM MgCl_2_ (from stock stored at 4°C, final 2 mM).
- Bring to 12 ml final volume with PBS^+^.

Heat the 4% paraformaldehyde at 37°C, add to the cells and continue fixation for 15 min at RT. Wash twice with PBS^+^. The 4% paraformaldehyde solution left can be stored at 4°C and used for up to 7 days from preparation.

##### Immunofluorescence on interneurons plated on PLL and LN

– Remove culture medium from wells by using a P1000 pipette, then immediately add 1 ml of prewarmed (37°C) 4% paraformaldehyde.
– Fix 15 min at RT.
– Wash delicately twice with PBS^+^ (by using a P1000 pipette to slowly add solutions along the side of the wells). If necessary, the procedure can be interrupted at this point by storing the cells in PBS^+^ at 4°C.
– Prepare a humidified chamber as described above. From now on all procedures are at RT, unless specified differently.
– To permeabilize the membranes of the neurons, use tweezers to remove 13 mm coverslips from the wells, and place them with cells facing down on 30 μl drops of Permeabilization Buffer (2x Permeabilization Buffer diluted 1:1 with dd water) placed on parafilm in a humidified chamber; incubate 4 min at RT.
– Gently inject 200 μl of PBS under each coverslip to lift them, and move them back to the wells with tweezers. Wash once with PBS.
– Dilute primary antibodies as required in 1x goat serum dilution buffer (prepared by dilution of a 2x stock with dd water); place 30 μl drops of diluted antibodies on parafilm in humidified chamber.
– Remove excess buffer from each 13 mm coverslip and put it with cells facing the drop; incubate overnight at 4°C in the humidified chamber, or for the required time at RT (as required for the specific primary antibodies).
– Transfer coverslips to wells by gently injecting 200 μl of PBS under each coverslip to lift them, and move them back with tweezers to the wells.
– Wash 3 times for 10 min each at RT with 1 ml/well of high salt solution.
– Prepare dilutions of secondary antibodies and fluorescent phalloidin (if required), by diluting fluorescent probes in 1x goat serum dilution buffer; if necessary, for nuclear staining dilute in the same solution also DAPI (stock solution diluted 1:500).
– Remove excess buffer from each 13 mm coverslip and put it with cells facing a 30 μl drop of diluted fluorescent probes in the humidified chamber; incubate 1 h at RT.
– Transfer coverslips to wells by gently injecting 200 μl of PBS under each coverslip to lift them, and move them back with tweezers to the wells.
– Wash once for 10 min with 1 ml high salt solution, then wash once for 5 min with PBS.
– Mount coverslips on glass slides by using Dako Fluorescent Mounting Medium and let dry for at least 2 h at RT.
– If the anti-bleaching solution with glycerol is used, pay attention not to use the oxidized solution (see preparation procedure above); with this medium the excess liquid has to be removed after mounting the coverslip on the slide, and the edge of the coverslip heed to be sealed with nail polish. Wait until sealing is dry.
– Store coverslips at −20°C.

##### Immunofluorescence on cells plated on matrigel–coated coverslips

– Immunofluorescence on Matrigel–coated coverslips is carried out avoiding to flip the coverslip over the drop of diluted antibodies, since this procedure may disrupt the coating gel, causing detachment of the Matrigel with cells from the glass. Instead, 50 μl of diluted antibodies are gently added on the coverslip placed on parafilm in a humid chamber, with cells facing up. The procedure works well for incubation with antibodies for up to 4 h at RT, or for up to 24 h at 4°C.
– After removing the antibodies with a yellow tip, cells are washed by delicately adding 200 μl of the required solution on each coverslip, and removing them with a yellow tip.
– Secondary antibodies and following washes are added and removed as the primary antibodies.
– Solutions and number of washings are the same as described for *Immunofluorescence on interneurons plated on PLL and LN*.
– After the final wash, excess liquid is removed, and coverslips are mounted as described for cells on PLL and LN.

**Figure 3:**
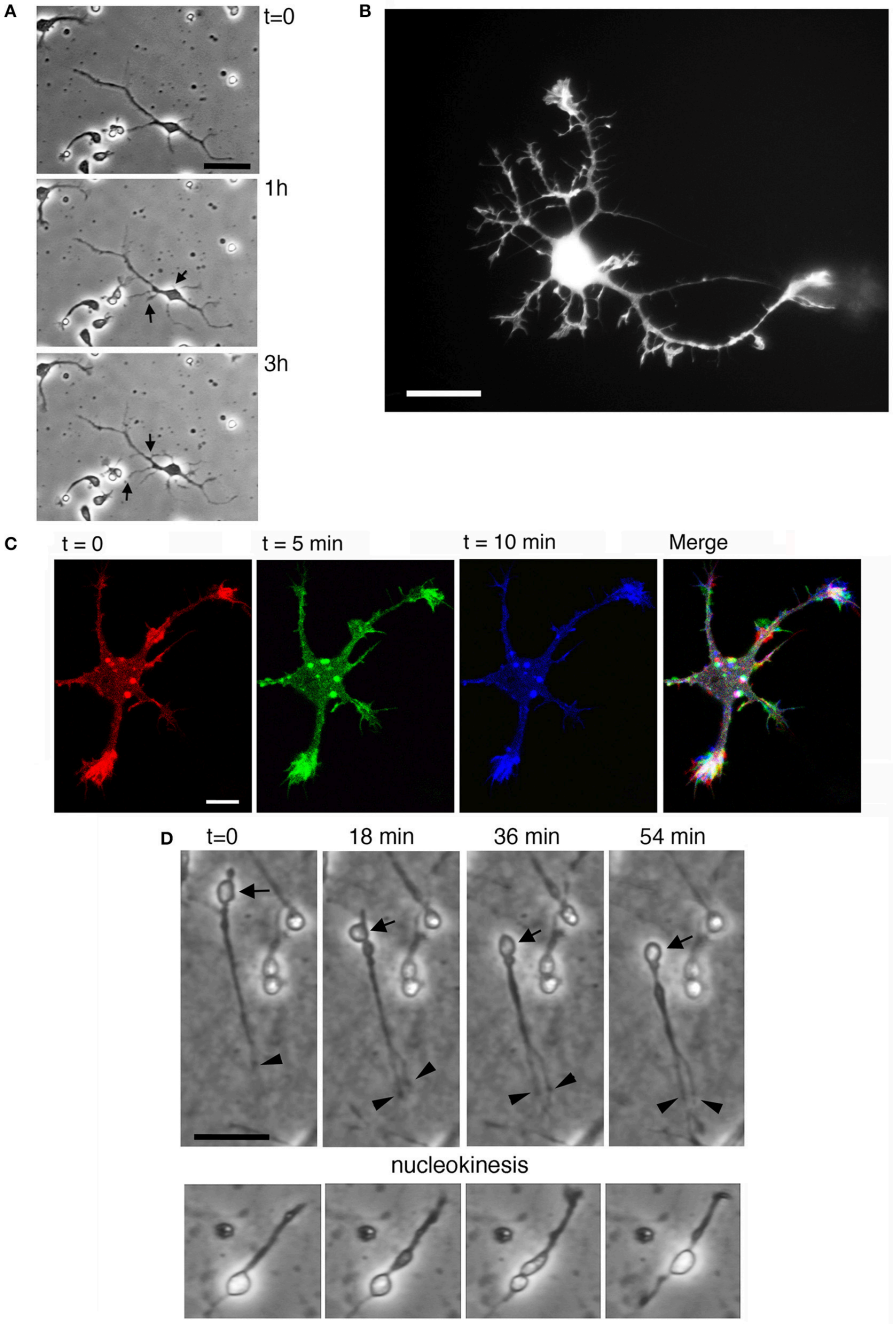
Characterization of MGE–derived cultures. **(A)** Frames (from Supplementary Movie 1) of live MGE–derived cells (50,000 cells/coverslip) imaged at DIV2. Bar, 25 μm. **(B)** DIV6 MGE–derived cell transfected with GFP-LifeAct. Bar, 20 μm. **(C)** Three confocal frames and their color-coded merge from a time-lapse of growth cones from a DIV5 interneuron expressing RFP-LifeAct. Bar, 10 μm. See also Supplementary Movie 1. **(D)** Frames from movies of live MGE–derived cells (50,000 cells/coverslip) imaged at DIV2. In the top series arrows point to a migrating cell, arrowheads to the tips of branches of the leading edge. Nucleokinesis is especially evident in the cell shown in the lower panel. Bars, 20 μm.

The development of neurites and the behavior of growth cones in dissociated MGE–derived cells on coated coverslips can be followed in living cells, for example at lower resolution by time-lapse analysis in phase contrast (Figure 3A; Supplementary Movie 1), or by higher resolution imaging (Figures 3B,C) after transfection of cells with cDNAs for the expression of fluorescently-tagged proteins (see below Transfection of MGE-derived cells). Interestingly, the migratory behavior of dissociated MGE–derived cells preserves the characteristics observed during the migration of these cells *in vivo*, including the nucleokinesis and the splitting of the leading process in branches that can be manipulated to modify their migratory trajectory (Figure 3D; Luxton et al., 2010; Marin et al., 2010).

#### Protocol for the preparation of lysates from MGE-derived cultures

##### Solutions

– TBS
– Thaw aliquots of the following stocks stored at −20°C: Complete (cocktail of anti-proteases, from Roche); 1 M sodium orthovanadate; 1 M sodium fluoride; 0.3 M PMSF.
– Prepare lysis buffer: 0.5% Triton X-100, 150 mM NaCl, 2 mM MgCl_2_, 20 mM Tris-HCl pH 7.5, 1 mM sodium orthovanadate, 10 mM sodium fluoride, 0.3 mM PMSF, 1x Complete.

##### Procedures

– All procedures are carried out at 0–4°C.
– Place on ice one or more 3.5 cm dishes with DIV2 MGE-derived cells isolated from E14.5 embryos.
– Remove medium by aspiration, and wash cells twice with TBS. After the last wash remove well excess liquid and add 30 μl lysis buffer. Use precooled scraper to collect lysate and transfer to precooled 1.5 ml Eppendorf tube. Mix with rotation for 15 min at 4°C. Spin 10 min at 18,000 g_max_ at 4°C. Transfer the supernatant in precooled Eppendorf tube. If not used immediately, the lysates can be frozen quickly and stored at −80°C.

**Figure 4:**
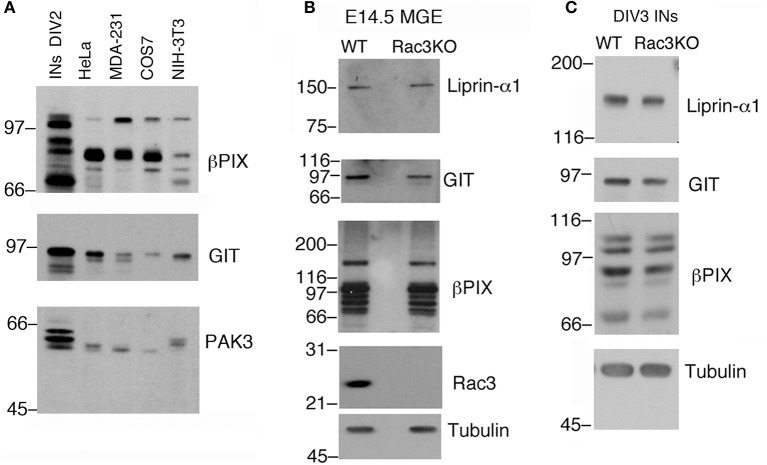
Immunochemical analysis on MGEs and MGE–derived cultures. **(A)** Immunoblotting to compare the expression of endogenous PIX, GIT, and PAK3 proteins in lysates of DIV2 MGE-derived cells, NIH-3T3, COS7, MDA-MB-231, and HeLa cells. Each lane was loaded with 100 μg of protein lysate. Filters were incubated with Abs for the indicated antigens. **(B)** Blots for comparison of protein expression in E14.5 MGEs isolated from WT or Rac3KO mice (60 μg protein lysate per lane). **(C)** Blots for comparison of protein expression in DIV3 dissociated cell cultures from E14.5 MGEs isolated from WT or Rac3KO mice (50 μg of protein lysate per lane).

Typically, 1.5–3 × 10^6^ dissociated E14.5 MGE–derived cells are plated in each 3.5 cm dish. As an example, analysis by immunoblotting revealed that proteins required for cell and growth cone motility are highly expressed in DIV2 MGE–derived cells compared to a number of different non-neuronal cell lines (Figure 4A). The possibility to collect enough cells to perform immunochemistry allows to compare protein expression in cell cultures with protein expression in isolated intact MGEs from wildtype and knockout mice (Figure 4B), and also to compare cultures from mice with different genotypes (Figure 4C).

**Figure 5:**
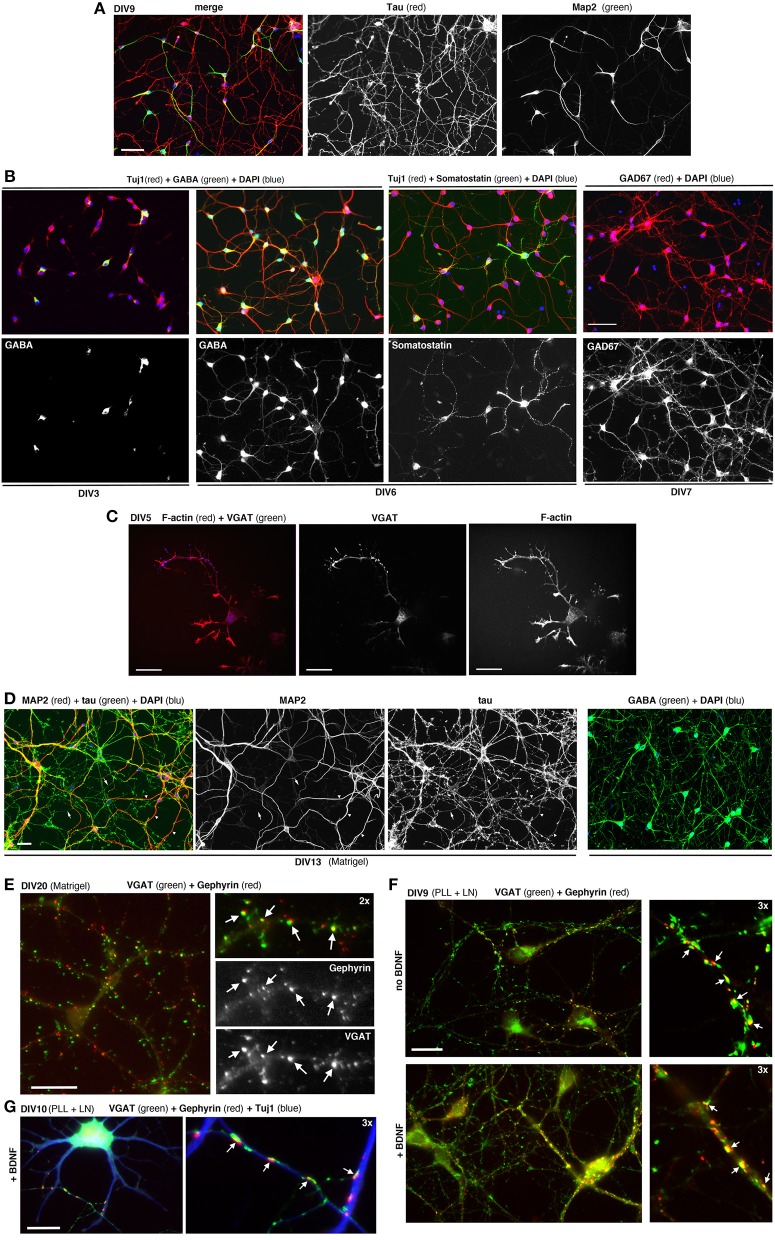
Dissociated MGE–derived cells develop into GABAergic interneurons. **(A)** DIV9 cells stained for axonal marker Tau and dendritic marker MAP2. **(B,C)** Cells at the indicated DIVs immunostained for different markers of GABAergic interneurons. **(D)** Left: DIV13 MGE–derived cells cultured on Matrigel were stained for Tau and MAP2. Examples of Tau–positive axons (arrows) and MAP2-positive dendrites (arrowheads) are shown. Right: DIV15 cells on Matrigel immunostained for GABA and DAPI. **(E)** DIV20 interneurons on Matrigel immunostained for GABAergic presynaptic terminals (VGAT) and inhibitory postsynaptic terminals (gephyrin). Arrows indicate sites of juxtaposition of the two markers. **(F,G)** DIV9 **(F)**, and DIV10 **(G)** cells cultured on PLL and LN with or without BDNF. Arrows in **(E–G)** indicate examples of juxtaposition of VGAT and gephyrin. Bars: 60 μm **(A,B)**; 40 μm **(D)**; 20 μm **(C,E,F)**; 12 μm **(G)**.

MGE–derived cells cultured on PLL and LN with or without BDNF form morphological synapses defined by juxtaposition of the pre- and postsynaptic markers VGAT and gephyrin (Sassoè-Pognetto et al., 2000), respectively. Synapses can be observed under these conditions at DIV9-DIV10 (Figure 5E). Generally at later time-points neurons tend to form clusters.

### Development of interneurons in cultures

MGE–derived cells plated on PLL and LN induce the formation of neurites that can be followed in time by analyzing cells fixed at different DIVs (Franchi et al., 2016). Once they have migrated to their final destination in the cortex and hippocampus, the differentiation program of interneurons involves the outgrowth and patterning of their dendritic and axonal arbors. The development of dissociated MGE–derived cells *in vitro* results in the polarized expression of the axonal marker Tau in the long and branched axon, and of MAP2 restricted to the soma and dendrites (Figure 5A). Most cells become GABAergic already by DIV6/DIV7, as determined by their positivity for GABA and the GAD67 isoform of the enzyme glutamic acid decarboxylase (GAD) that catalyzes the decarboxylation of glutamate to GABA, and is diffused in the cell (Figure 5B). At DIV6 some cells already show markers for specific subtypes of MGE–derived interneurons, as shown for somatostatin. In developing interneurons the presynaptic marker VGAT (vesicular GABA transporter) is found concentrated along the longest neurite, as expected for the accumulation of this transporter during the development of the axonal presynaptic terminals (Figure 5C). In longer term cultures on Matrigel (2.5 mg/ml) a dense neuritic network including Tau-positive axons and MAP2-positive dendrites is observed, with virtually all cells expressing high levels of GABA (Figure 5D). Neurons on Matrigel develop morphological synapses identified by the juxtaposition of the axonal presynaptic marker VGAT and the postsynaptic marker gephyrin (Figures 5E–G), a major scaffold protein of inhibitory synapses linking postsynaptic receptors including GABAergic receptors to the cytoskeleton (Tyagarajan and Fritschy, 2014). Although Matrigel has shown to be a more reliable substrate for long-term cultures compared to PLL and LN (tested up to 20 DIVs), morphological synapses can be detected also in cultures on PLL and LN already by DIV9, suggesting that this substrate may induce a relatively rapid development of GABAergic cells in culture.

### BDNF improves the development of neurites in dissociated MGE–derived cells

We observed a certain degree of variability in the survival of the dissociated cells among different preparations, especially after transfection (see next sections). The neurotrophin BDNF (brain derived neurotrophic factor) is known to promote neuritogenesis in neocortical interneurons (Jin et al., 2003), and cell viability, neuritic branching, and the development of the GABAergic phenotype in MGE-derived cultures (Pozas and Ibáñez, 2005). We therefore tested the effects of BDNF on the cultures by quantifying its effects on neuritic development. BDNF was added at 50 ng/ml to DIV1 cultures and kept until fixation of cells at DIV6. Qualitative observation of several cultures indicated that BDNF improved the reproducibility of MGE–derived cultures. Moreover, BDNF visibly enhanced the growth of neurites.

**Figure 6:**
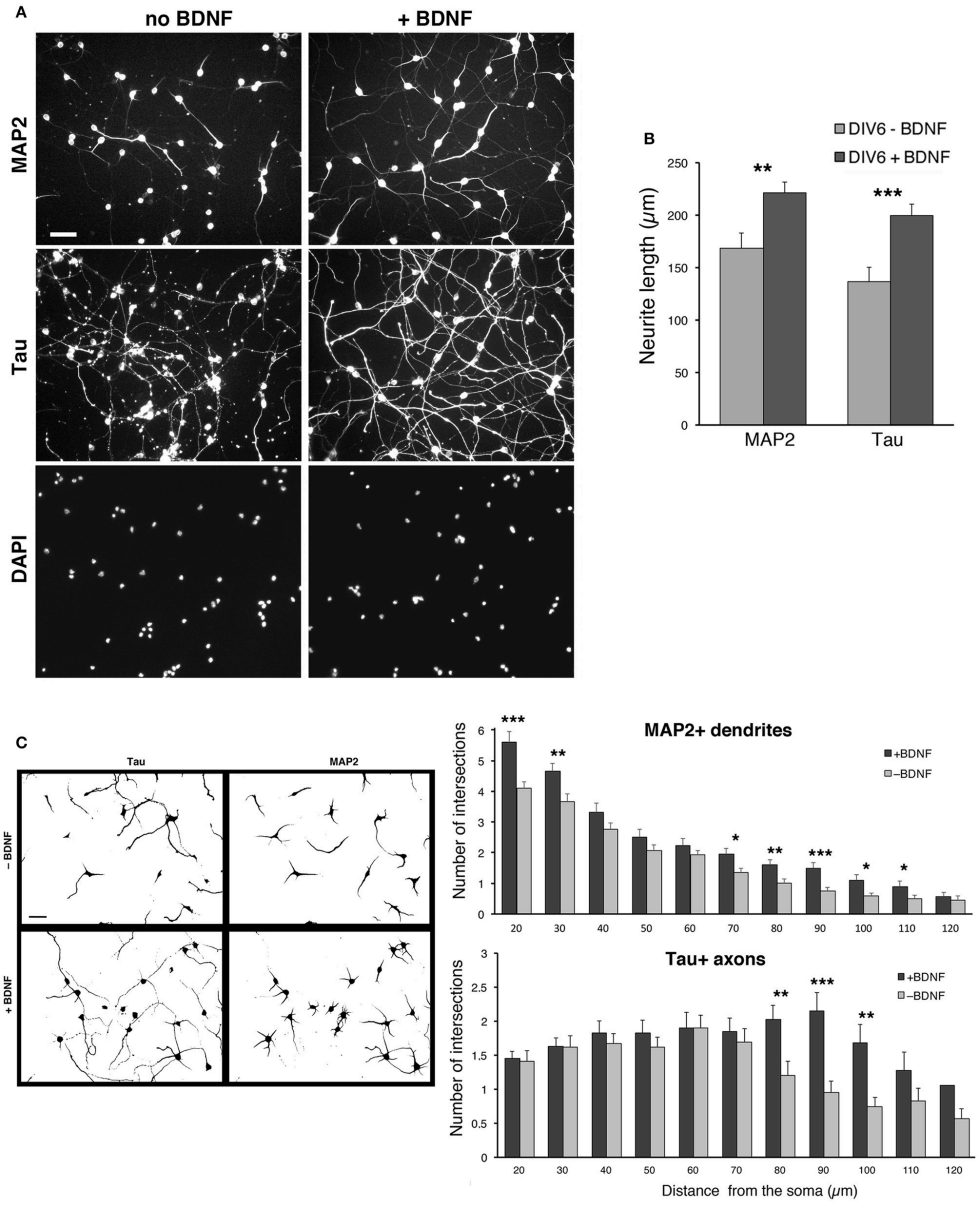
BDNF promotes neurite outgrowth from dissociated MGE–derived cells. **(A)** DIV6 neurons incubated without or with BDNF (50 ng/ml) and immunostained for MAP2 and Tau. Nuclei are revealed by DAPI staining. Each column shows the three stainings of the same field. Bar, 40 μm. **(B)** Quantification of the total length of neurites per cell. Bars are mean values of the average total length of MAP2-positive and Tau-positive neurites per cell from DIV6 cultures. Bars are mean values ± SEM from 20 fields per experimental condition, with a total of 285 BDNF–treated cells, and 308 untreated cells. ^**^p = 0.00525 (MAP2-positive neurites); ^***^p = 0.00083 (Tau-positive neurites). **(C)** Left: examples of inverted images of DIV6 MGE–derived cells cultured with or without BDNF. Bar, 40 μm. Right: Sholl analysis on DIV6 MGE–derived neurons cultured with ±50 ng/ml BDNF; bars are mean values ±SEM from 17 cells per experimental condition. ^*^p < 0.05; ^**^p < 0.01; ^***^p < 0.001.

In cultures without BDNF the staining for MAP2 was less pronounced, and the staining for Tau showed axons that appeared less preserved and developed compared to cultures with BDNF (Figure 6A). Cultures with 50 ng/ml BDNF showed morphologically preserved neurons with stronger MAP2 signal and intact, more extended Tau1-positive axons. No evident difference could be detected between cultures with 50 or 100 ng/ml BDNF. Therefore, 50 ng/ml BDNF added at DIV1 is chosen as the standard condition for culturing MGE-derived cells. Quantification shows that both the total extension of MAP2-positive and Tau-positive neurites is significantly increased in BDNF-treated cells compared to control untreated cells (Figure 6B). Sholl analysis has been used to quantify dendritic (MAP2-positive) and axonal (Tau-positive) branching in DIV6 cells immunostained with anti-MAP2 and anti-Tau Abs. Sholl analysis confirmed the increased number of MAP2-positive dendrites per cell and Tau-positive axonal branching (Figure 6C).

**Figure 7:**
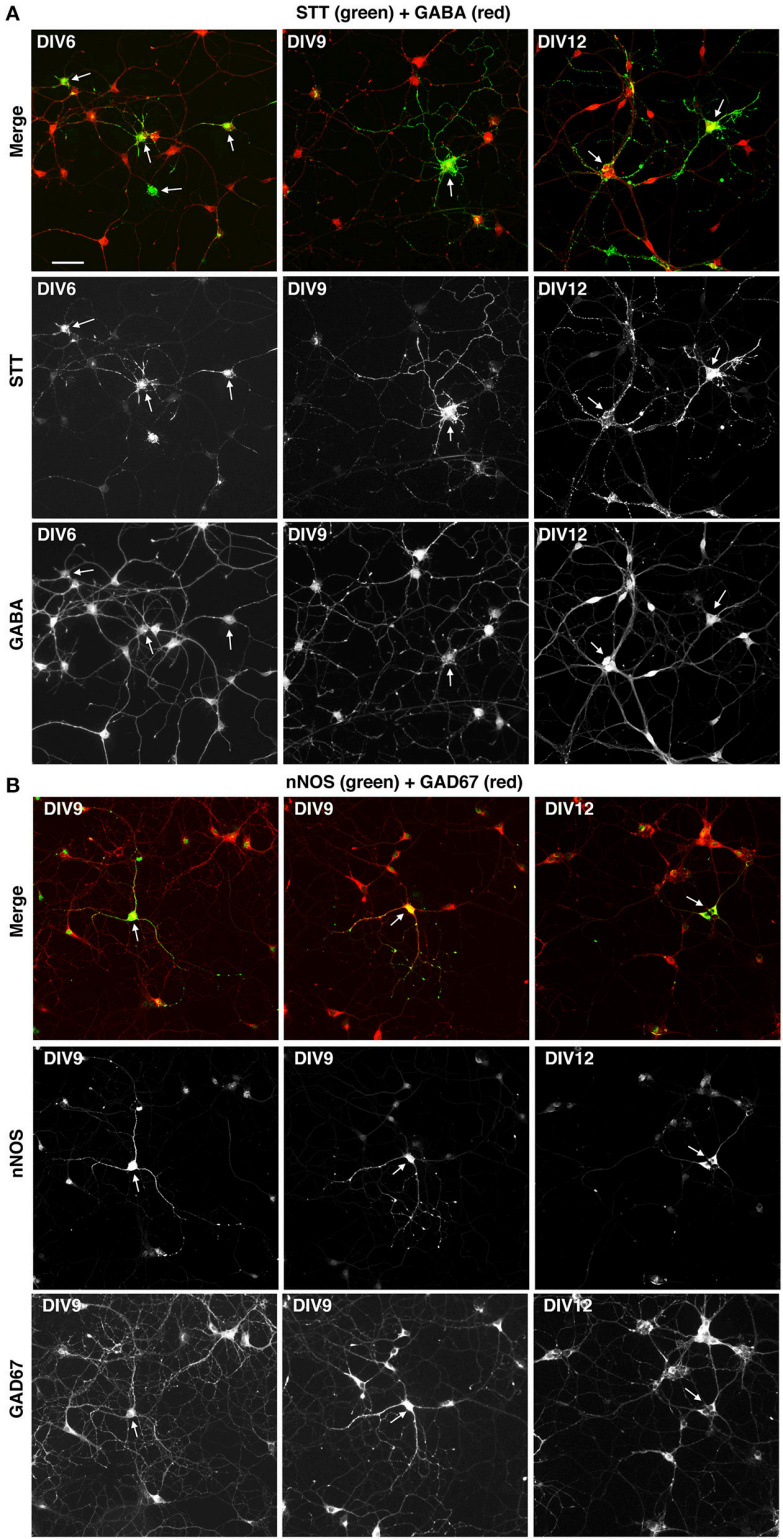
Expression of markers for different types of interneurons in MGE-derived cultures. MGE-derived cells were plated on PLL- and LN-coated coverslips and cultured up to DIV6–DIV12 before fixation for immunostaining with the indicated antibodies. **(A)** A subset of GABA-positive cells are STT-positive (arrows). **(B)** Some GAD67-positive cells are nNOS-positive (arrows). Bar, 50 μm.

We next tested if the described culture system allowed the development *in vitro* of specific GABAergic subtypes. For this we analyzed MGE–derived cells plated on glass coverslips coated with PLL and LN, and cultured with BDNF for different times in culture. The cultures were analyzed by immunofluorescence with Abs against neuronal nitric oxide synthase (nNOS), parvalbumin (PV), and somatostatin (STT), since cortical interneurons expressing these markers are expected to originate largely from the MGE, and with anti-calretinin antibodies that should label interneurons derived from the caudal ganglionic eminences (Wonders and Anderson, 2006; Tricoire et al., 2011; Inan et al., 2012). We observed a strong specific signal in these cultures with the anti-somatostatin Ab, which detected somatostatin-positive GABAergic neurons already in DIV6 (Figure 7A). Positive cells were detected also with anti-nNOS (Figure 7B), while anti-PV Abs gave quite a high non-specific background in these cultures, like the anti-CR Ab (not shown). These results indicate that different GABAergic subtypes can develop in these MGE-derived cultures.

### Set up of transfection of MGE-derived cells

One advantage of using MGE-derived cells is the possibility to transfect developing interneurons with plasmids to address the function of endogenous or overexpressed proteins during the development of GABAergic cells. Various conditions were tested to optimize the protocol for the transfection of dissociated MGE-derived cells.

We tested different electroporation programs with an AMAXA electroporator with dissociated MGE–derived cells before plating (DIV0). In the best condition among those tested, using 10^6^ cells and 1 μg DNA per transfection, the efficiency of transfection after electroporation was 16% (±2.1 SEM; *n* = 10 fields per condition) at DIV2. The apparent increase of efficiency at DIV3 and DIV7 cultures (18% ± 2.1 and 24% ± 3.3, respectively) was indeed accompanied by a clear increase in cell loss. Moreover, the levels of expression of transfected GFP were lower than in cells transfected with lipophilic agents, and transfected cells appeared poorly developed in terms of neurites (not shown).

Next, transfection on adherent cells was tested using lipophilic reagents with plasmids for either GFP or GFP-LifeAct. We compared the use of Effectene (Qiagen) and Lipofectamine-2000 (Thermo Fisher Scientific). Transfection with either 0.1 or 0.2 μg DNA with 0.6 μl Effectene for 4 h at DIV1 resulted in low toxicity but also low efficiency of transfection at DIV6. No transfected cells were detected after transfection with Effectene at DIV3. Transfection with Lipofectamine-2000 gave better results, with higher transfection efficiency, although it may result in more evident effects on the cultures, as detected by the tendency of the cells to form clusters. Transfection with Lipofectamine at DIV1 generally resulted in a good transfection of the cells. We therefore set to optimize the conditions for transfection with Lipofectamine-2000 by considering different parameters: cell density; substrate for attachment; time of transfection; amounts and ratio between DNA and transfectant in the transfection mix; incubation time with transfection mix; use of BDNF after transfection. Analysis of the transfected cultures was generally performed at DIV4-DIV6. 100,000–120,000 dissociated cells per 13 mm diameter coverslip were plated in 24-well dish.

Cells were tested for transfection at DIV1. We tried different combinations of DNA (pEGFP-N1 plasmid) and Lipofectamine-2000: either 0.1 or 0.2 μg of DNA were combined with either 0.35 or 0.5 μl of Lipofectamine-2000. We plated 100,000 MGE-derived cells per well (24 wells plate) with coverslips coated with 200 μg/ml PLL and 20 μg/ml LN. Cells were lipofected at DIV1 and fixed at either DIV4 or DIV6 to evaluate transfection efficiency. Transfection was evaluated by looking at the presence and morphology of GFP-positive cells, considering the formation of neurites by transfected cells, and evaluating the preservation of the network of non-transfected neurons detected by immunostaining with the mAb TuJ1 recognizing neuronal tubulin β3 or with anti-MAP2 Abs.

**Figure 8:**
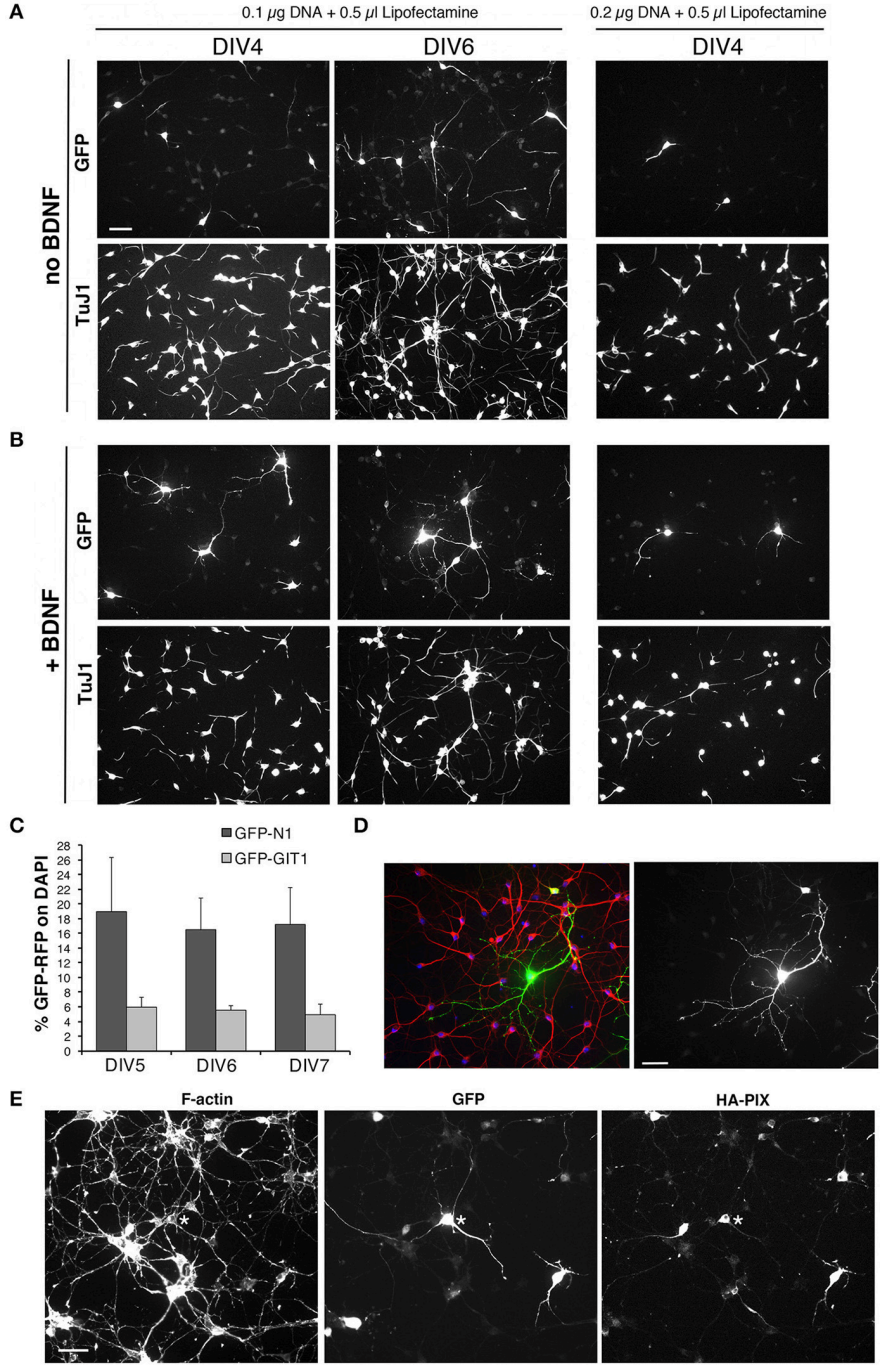
Setup of transfections of MGE-derived cells. **(A,B)** Test of different conditions of MGE–derived cells plated on 200 μg/ml PLL and 20 μg/ml LN, transfected at DIV1, and fixed at either DIV4 or DIV6 for immunofluorescence. After transfection cells were cultured without **(A)** or with 50 ng/ml BDNF **(B)**. **(C)** Quantification of the percentage of cells transfected at DIV1 with 0.1 μg of the indicated plasmid DNA and 0.5 μl of Lipofectamine-200, and fixed at different DIVs for immunofluorescence. Bars are means ±SEM of percentage of DAPI-positive cells expressing GFP. **(D)** Immunofluorescence of a culture transfected with GFP grown in the presence of BDNF, stained with Abs for GFP (green), for MAP2 (red), and with DAPI (blue). **(E)** DIV6 cells cotrasfected at DIV1 with 0.05 μg of each plasmid (total of 0.1 μg of plasmid DNA), to express GFP and HA-tagged guanine nucleotide exchange factor PIX.

Transfection of primary neurons is expected to cause loss of cells, but a good transfection should result in the maintenance of a good neuronal network that is expected to help supporting neuronal survival and development. Almost no transfected cells were detectable at DIV4 in cultures transfected with either 0.1 μg or 0.2 μg DNA and 0.35 μl transfectant, while some transfected cells with good morphology were found under these transfection conditions in cultures fixed at DIV6 (not shown). Several transfected cells were observed both at DIV4 and DIV6 in cultures transfected with 0.1 μg DNA and 0.5 μl lipofetamine, while very few cells with poor morphology were visible in samples transfected with 0.2 μg DNA and 0.5 μl Lipofectamine (Figure 8A). Under these conditions, the network of non-transfected cells was also more strongly affected compared to cultures transfected with 0.1 μg DNA and 0.5 μl Lipofectamine. The addition of 50 ng/ml DBNF after transfection at DIV1 resulted in more consistent results in terms of preservation of the neuronal network and development of neurites in DIV4 and DIV6 cultures transfected with 0.1 μg DNA and 0.5 μl Lipofectamine. While neuronal morphology remained poorer in cultures transfected with 0.2 μg DNA and 0.5 μl Lipofectamine (Figure 8B).

In conclusion, optimal conditions for transfection and immunofluorescence on MGE–derived cells were observed by plating 100,000–120,000 cells on glass coverslips coated with 200 μg/ml PLL and 20 μg/ml LN, transfecting at DIV1 with 0.1 μg of DNA and 0.5 μl of Lipofectamine-2000, in the presence of 50 ng/ml BDNF added after transfection at DIV1.

To check whether these were the optimal conditions for coating with PLL and LN, we went back trying six different conditions obtained by coating the coverslips with solutions of either 100, 200, or 500 μg/ml PLL, followed by coating with either 20 or 40 μg/ml LN. At DIV1 cells on the different substrates were transfected with the pEGFP-N1 plasmid, and cells were then fixed at DIV4, DIV5, and DIV6 for analysis. Qualitative morphological analysis of the network confirmed that the combination of 200 μg/ml PLL with 20 μg/ml LN represented an optimal condition to obtain good transfection with nice neuronal morphology.

Although some variability was observed among different experiments, transfection with the plasmid pEGFP-N1 for GFP ranged between 7.5% ± 2.1 to 16.5 ± 4.3 (means ± SEM; *n* = 4–10 fields) of transfected cells at DIV6. For larger proteins, the percentage of transfected cells was generally lower: as one example, transfection with the plasmid pEGFP-GIT1 for the expression of the GFP-GIT1 (110 kDa) gave a transfection efficiency of 5.6% ± 0.6 (mean ±SEM; *n* = 5 fields; Figure 8C). While these transfection efficiencies are not suitable for biochemical analysis, they are optimal for morpho-functional analysis, since they allow to isolate the transfected cell from the dense network of non-transfected cells present in the cultures (Figure 8D). The conditions established may be used either to overexpress proteins or protein fragments, or to transfect plasmids for shRNA to obtain protein silencing.

Starting from the conditions established for the transfection of a single plasmid, we have tested co-transfection to allow the coexpression of two different proteins, but also to silence one endogenous protein by shRNA and at the same time overexpress a protein. We found that a good cotransfection was achieved with 0.1 μg of total DNA (0.05 μg per plasmid) and 0.5 μl of Lipofectamine-2000 (Figure 8E).

#### Protocol for the transfection of dissociated MGE–derived cells

– MGE-derived cells are seeded at 100,000–120,000 cells per well on glass coverslips placed in 24-well culture dishes, with 0.6–0.8 ml of maintenance medium per well.
– Transfection is performed at DIV1.
– For each well prepare in separate tubes: 0.1 μg of DNA in 50 μl of Optimem/well, and 0.5 μl of Lipofectamine-2000 in 50 μl Optimem/well; incubated the two solutions separately for 5 min a RT. For co-transfection of two plasmids, the same total amount of DNA (in a final volume of 50 μl) and Lipofectamine-200 (in a final volume of 50 μl) is used. A first test can be done by using 0.05 μg of DNA for each plasmid. If one plasmid is transfected more easily that the other, relative amounts can be adjusted in favor of the less efficient plasmid, keeping the total of 0.1 μg of DNA.
– DNA and Lipofectamine solutions are then mixed, and this transfection mix is incubated 20 min at RT.
– 200 μl of maintenance medium are left in each well (400-600 μl are removed from each well, pooled, and stored in a sterile 15 ml tube at 37°C).
– Add 100 μl/well of transfection mix to the 200 μl of maintenance medium left in each well, and incubate cells for 20 min in 37°C incubator (see note below).
– Remove the transfection medium from wells, and add back 400 μl of conditioned maintenance medium previously collected from the cultures, to which BDNF to a final concentration of 50 ng/ml has been added. To avoid drying of the coverslips, to change the medium remove it with a P1000 pipette from 1–3 wells at a time.

Note: one effect of the incubation of the cells with the transfection mix is their partial detachment and formation of aggregates (Supplementary Figure 2). Tendency to aggregation during transfection can be decreased by shortening the incubation time of cells with the transfection mix. On the other hand, if the transfection efficiency is poor and the neuronal network still well-preserved, the time of transfection can be extended from 20 min up to 45 min. Given the complexity of the procedure, variability with optimal transfection times (between 20 and 45 min) has been observed by different operators.

### Effects of gene deletion on migration of MGE–derived cells

**Figure 9:**
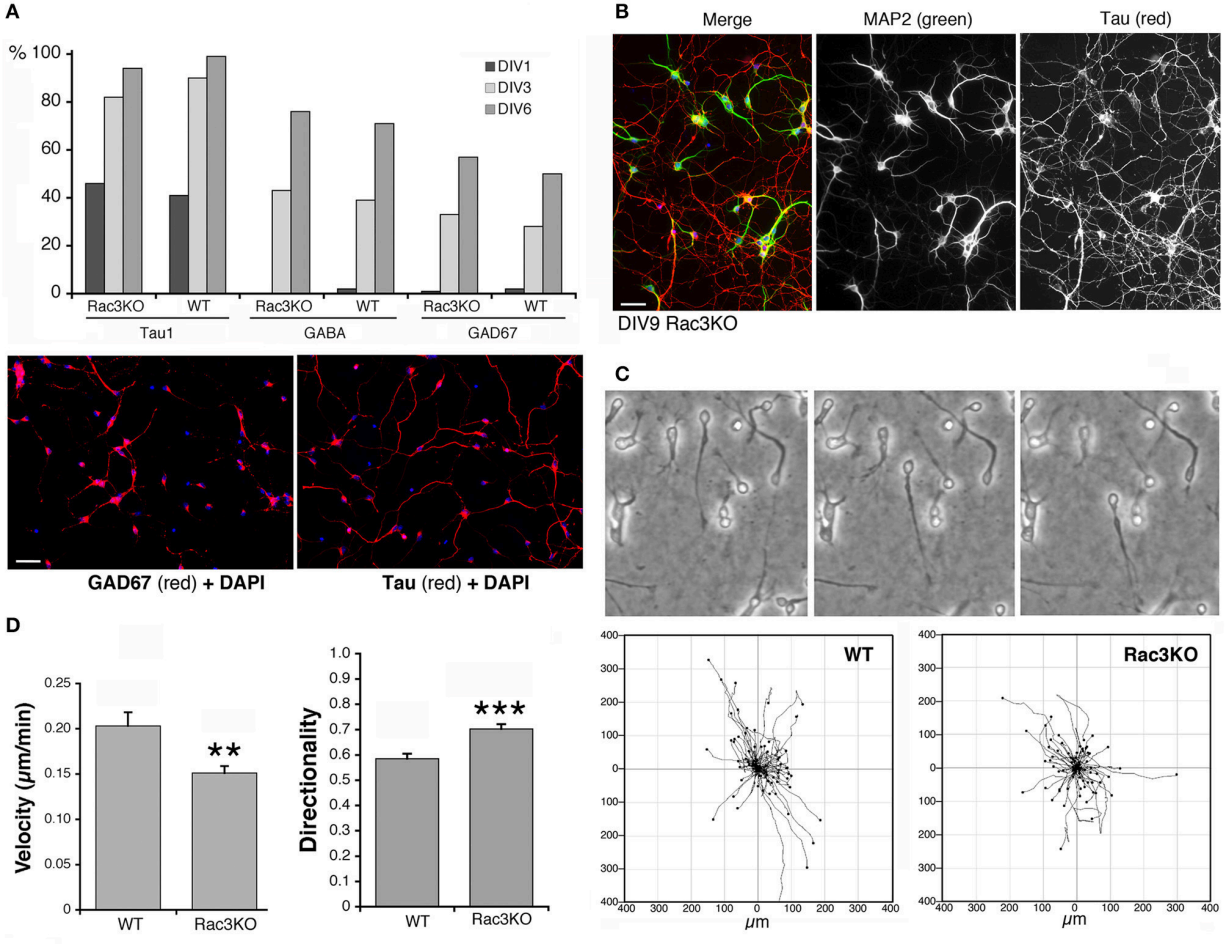
Comparison of Rac3KO and WT MGE-derived cells. **(A)** Top panel: quantification of the expression of neuronal and GABAergic markers. The analysis was performed on images acquired on DIV1, DIV3, and DIV6 cultures of MGE-derived cells from E14.5 WT or Rac3KO mice immunostained for the axonal marker Tau, and the GABAergic markers GABA and GAD67. The graph shows the percentage of MGE-derived cells positive for the indicated markers quantified at DIV1, DIV3, and DIV6. The percentage of positive cells for each marker was calculated as the ratio between the number of marker-positive cells × 100/the number of DAPI-positive nuclei (*n* = 566–1620 cells per marker per genotype). Lower panel: examples of DIV6 MGE-derived cells immunostained for GAD67 (left) and Tau (right); DAPI in blue. Bar, 40 μm. **(B)** DIV9 MGE–derived cells immunostained for MAP2 (green) and Tau (red); DAPI staining in blue. Bar, 40 μm. **(C,D)** Analysis of cell motility reveals defects in Rac3KO random migration. MGE–derived cells from WT and Rac3KO E14.5 mice were plated on Matrigel–coated glass coverslips (2.5 mg/ml), and imaged at DIV2 for 14 h. **(C)** Top: examples of migrating WT cells from time-lapses. Bottom: plots of cell motility from time-lapses (*n* = 120 cells per plot). See also Supplementary Movies 2, 3. **(D)** Quantification of cell motility: bars are means ±SEM; *n* = 223 motile cells per genotype, from three different experiments. ^**^p = 0.00214 (velocity); ^***^ *p* = 0.00002 (directionality).

Rac1 and Rac3 GTPases are important for the development of functional MGE-derived cortical/hippocampal GABAergic interneurons (de Curtis, 2014; Vaghi et al., 2014; Tivodar et al., 2015), and deletion of either GTPase negatively affects GABAergic synapses, with cognitive/behavioral consequences and impairment of inhibitory circuits (Pennucci et al., 2016). Here, we have used the comparison of MGE–derived cells from WT vs. Rac3KO mice as one example of the use of the MGE–derived culture system to address the behavior of interneurons from mutant mice *in vitro*. The Rac3 GTPase is increasingly expressed in developing MGE–derived interneurons *in vitro* (Franchi et al., 2016), and analysis *in vivo* of Rac3KO mice has revealed specific defects in the development of GABAergic cells as well as behavioral phenotypes (Corbetta et al., 2008; Pennucci et al., 2016). Moreover, Sholl analysis on dissociated Rac3KO cells revealed that the endogenous Rac3 protein promotes the development of the neuritic tree of MGE–derived cells in culture (Franchi et al., 2016). On the other hand, Rac3KO cells do not show defects in the differentiation of the GABAergic phenotype *in vitro*, since the percentage of cells positive for the axonal marker Tau and for the GABAergic markers GABA and GAD67 were similar in WT and Rac3KO cells (Figure 9A). In both WT and Rac3KO cells BDNF did not affect the percentage of cells with neurites positive for axonal and dendritic markers Tau and MAP2. Moreover, Rac3KO cells polarized normally, since at DIV9 they show a distinct Tau-positive axonal network and MAP2-positive dendrites (Figure 9B).

We have then tested the effects of Rac3 KO on cell migration, by comparing the motility of dissociated DIV2 cells derived from Rac3KO and WT mice on Matrigel–coated coverslips (Supplementary Movies 2, 3; Figure 9C). Quantification showed that while the fraction of motile cells was similar in WT (63% motile cells; *n* = 355) and Rac3KO (67% of motile cells; *n* = 355) MGE-derived cultures, their migratory behavior *in vitro* was different. Rac3KO cells showed reduced velocity and increased directionality compared to WT cells (Figure 9D). These results support the hypothesis that Rac3 promotes together with Rac1 the migration of MGE–derived precursors, and may explain the reduction in MGE–derived interneurons observed in the cortex and hippocampus of Rac3KO mice (Vaghi et al., 2014). Moreover, the results represent a proof of principle for the use of these cultures for the analysis of the migration of GABAergic precursors *in vitro*.

## Conclusion

Understanding the mechanisms that guide interneuron development and maturation represents a central aspect of the current research on cortical/hippocampal GABAergic interneurons. This type of analysis is highly relevant to brain function and to the pathology of various neuropsychiatric diseases. In this methodological study we have described the setting up and optimization of primary cultures obtained by dissociation of mouse embryonic MGEs. Most cells in these cultures develop *in vitro* into GABAergic interneurons, which can be reproducibly obtained by the set of protocols that we have carefully tested and described in details. Although short-term MGE-derived cultures have been used before (Pozas and Ibáñez, 2005; Cobos et al., 2007; Zimmer et al., 2008; Rudolph et al., 2010; Li et al., 2012; Villar-Cerviño et al., 2015), this is to our knowledge the first detailed description of the culture and transfection conditions for primary murine interneurons, including conditions that allow both short-term cultures for the analysis of the migration of the precursors, as well as long-term cultures. We have shown that in these cultures MGE-derived cells may develop into polarized GABAergic interneurons and can form morphologically identifiable inhibitory synapses.

Optimal conditions have been achieved by combining the use of proper extracellular substrates, with the use of BDNF to make the development of these cells *in vitro* more consistent. Moreover, we have established conditions to transfect the dissociated MGE–derived cells for protein expression or downregulation. As one example, we have also shown here the comparison of some of the properties of cultures from dissociated MGEs from WT vs. Rac3KO mice.

To the best of our knowledge, no refined description is available in the literature of this type of cultures, which present several advantages for the analysis of interneuron development *in vitro* with respect, for example, to the mixed cortical or hippocampal cultures often used in the literature. The availability of cultures of interneurons allows to address both the extracellular and intracellular mechanisms driving the development and maturation of these cells in a simplified system, in the absence of the complex intercellular interactions present *in vivo*. For example, it is possible to compare the development of neurites and inhibitory synapses by isolating the precursors from the MGE of WT vs. mutant animals. This is an extremely important aspect to complete and expand the analysis of the phenotypes of relevant transgenic/KO animal models, also considering the high relevance of interneuronopathies in several neural and psychiatric diseases.

Among their possible uses, the culture system described here has the potential to allow: (i) the identification of cell autonomous molecular mechanisms relevant to interneuron development and function; (ii) the comparative analysis of the development/maturation of WT vs. mutant interneurons from transgenic/knockout mice; (iii) the study of the effects of the expression of shRNAs and plasmids on different aspects of GABAergic cell development; (iv) reconstitution studies by adding interneurons to classical preparations of hippocampal or cortical neurons, starting from mice with different genotypes (e.g., WT interneurons and KO hippocampal neurons, or *vice versa*).

In conclusion, we believe that the detailed protocols described in this study may be useful to several neurobiologists interested in various aspects of interneurons biology, since they provide a powerful experimental tool to complement studies *in vivo* on the morpho-functional properties of GABAergic interneurons under physiological or pathological conditions.

## Ethics statement

This study was carried out in accordance with the recommendations of the national regulations (D.L. n 116, G.U. suppl. 40, 1992 February 18, circular Nr. 8, G.U., 1994 July 14) and with the international agreements in force (EEC Council directive 86/609, OJ L 358, 1 DEC 12, 1987; NIH Guide for the Care and use of Laboratory Animals, U.S. National Research Council, 1996). The protocol was approved by the IACUC of the San Raffaele Scientific Institute.

## Author contributions

SF, RM, VA, and IdC contributed to the conception and design of the work; SF, VA, RM, DT, ES, KS, and MB developed the experimental design; SF, FV, and IdC wrote the manuscript.

### Conflict of interest statement

The authors declare that the research was conducted in the absence of any commercial or financial relationships that could be construed as a potential conflict of interest.

## Supplementary material

Supplementary Figure 1 Aggregation assay; 400 μl aliquots with 300,000 dissociated MGE-derived cells resuspended in a filter-sterilized solution containing 125 mM NaCl, 2.5 mM KCl, 33 mM D-glucose, 2.6 mM CaCl2, 25 mM Hepes pH 7.4, were plated on coverslips coated with 2.5 mg/ml Matrigel, and incubated at 37°C on a rotatory shaker at 80 rpm. At the indicated times rotation was interrupted and images were taken by phase contrast at a Zeiss inverted microscope with 20 × lens. Bar, 100 μm.

Supplementary Figure 2 Transfection-induced cell aggregation. Cells were plated on coverslips coated with PLL and LN (120,000 cells/coverslip), and transfected at DIV1 with the transfection mix indicated on each panel. Incubation at 37°C with the transfection mix including 0.1 μg DNA and 0.5 μl Lipofectamine-2000 was for either 45 or 20 min. Cells were fixed at DIV6 to evaluate transfection and cell aggregation. In this test a decrease of aggregation with good transfection was observed after incubating cells for 20 min with the transfection mix. Bar, 40 μm.

Supplementary Movie 1 Time-lapse of a DIV5 MGE-derived cell plated on PLL and LN and transfected at DIV1 with RFP-LifeAct. Images for time-lapse were acquired with a confocal TCS SP8 microscope (Leica) equipped with 63 × 1.4 NA oil-immersion objective. During image acquisition cells were maintained in a temperature- and CO_2_-controlled chamber at 37°C.

Supplementary Movie 2 Time-lapse of DIV2 cells derived from MGE of WT mice plated on coverslips coated with Matrigel. Phase contrast images for time-lapse were acquired in a temperature- and CO_2_-controlled chamber at 37°C, at 9 min per frame, with a Zeiss Axiovert S100 microscope with 20 × objective.

Supplementary Movie 3 Time-lapse of DIV2 cells derived from MGE of Rac3KO mice plated on coverslips coated with Matrigel. Phase contrast images for time-lapse were acquired in a temperature- and CO_2_-controlled chamber at 37°C, at 9 min per frame, with a Zeiss Axiovert S100 microscope with 20 × objective.

## Abbreviations

DAPI: 4′,6-Diamidine-2′-phenylindole dihydrochloride
dd: double distilled
DIV: days *in vitro*
ECM: extracellular matrix
FN: fibronectin
GABA: γ-aminobutyric acid
GAD: glutamic acid decarboxylase
HBSS: Hanks’ Balanced Salt Solution
LN: laminin-1
MGEs: medial ganglionic eminences
PLL: poly-L-lysine
RT: room temperature.
