# Supplementary figures and images for: A Method to Culture GABAergic Interneurons Derived from the Medial Ganglionic Eminence
Source: Front Cell Neurosci. 2018 Jan 8;11:423. doi: 10.3389/fncel.2017.00423 (PMC5766683; doi:10.3389/fncel.2017.00423)

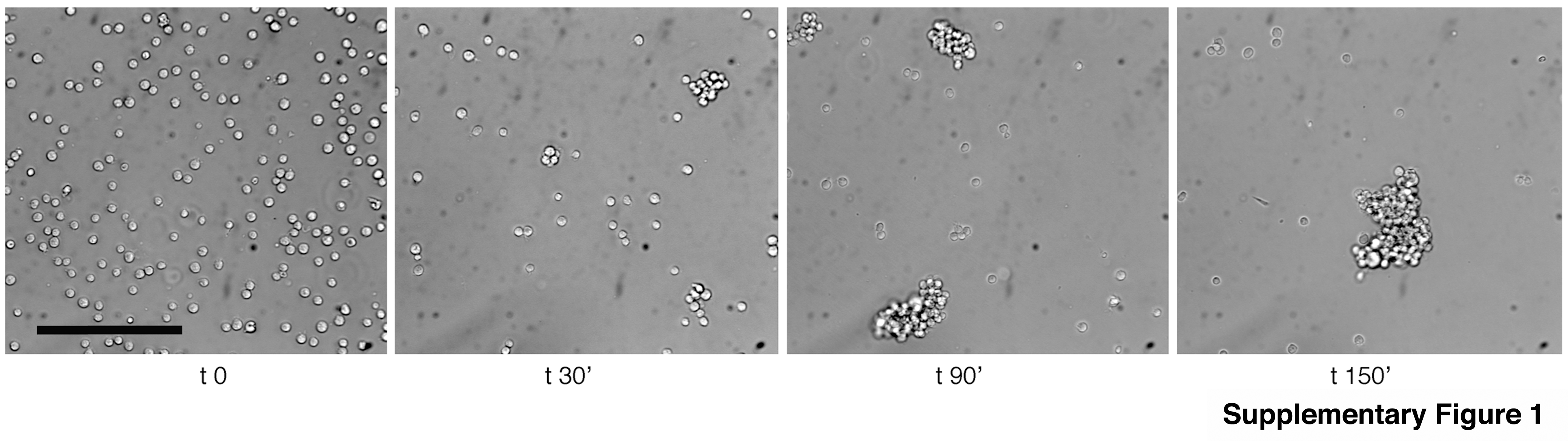

Supplement: Supplementary Figure 1 — Aggregation assay; 400 μl aliquots with 300,000 dissociated MGE-derived cells resuspended in a filter-sterilized solution containing 125 mM NaCl, 2.5 mM KCl, 33 mM D-glucose, 2.6 mM CaCl2, 25 mM Hepes pH 7.4, were plated on coverslips coated with 2.5 mg/ml Matrigel, and incubated at 37°C on a rotatory shaker at 80 rpm. At the indicated times rotation was interrupted and images were taken by phase contrast at a Zeiss inverted microscope with 20 × lens. Bar, 100 μm. [file Image1.TIF]

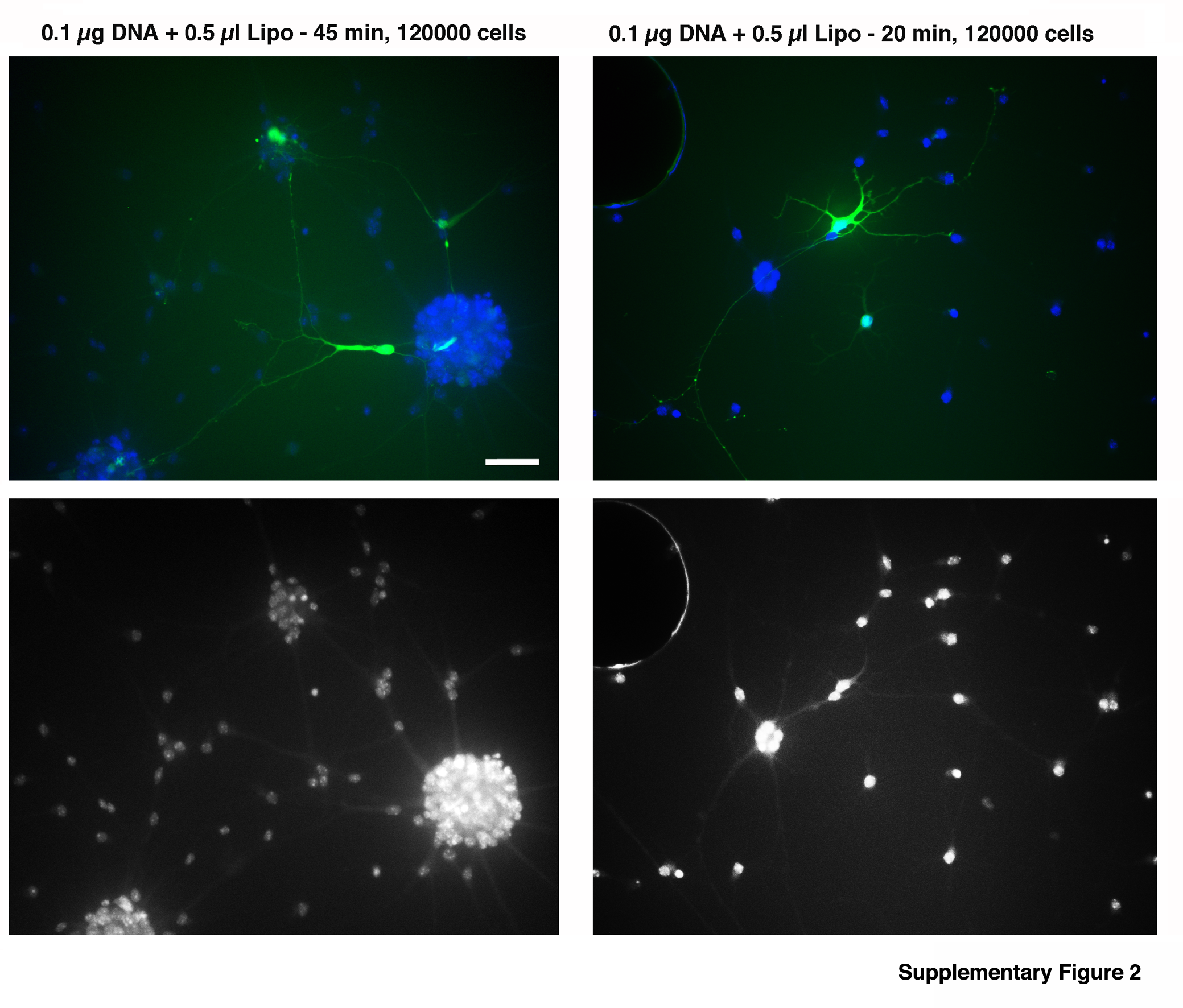

Supplement: Supplementary Figure 2 — Transfection-induced cell aggregation. Cells were plated on coverslips coated with PLL and LN (120,000 cells/coverslip), and transfected at DIV1 with the transfection mix indicated on each panel. Incubation at 37°C with the transfection mix including 0.1 μg DNA and 0.5 μl Lipofectamine-2000 was for either 45 or 20 min. Cells were fixed at DIV6 to evaluate transfection and cell aggregation. In this test a decrease of aggregation with good transfection was observed after incubating cells for 20 min with the transfection mix. Bar, 40 μm. [file Image2.TIF]
